# Synthesis and dissociation of soliton molecules in parallel optical-soliton reactors

**DOI:** 10.1038/s41377-021-00558-x

**Published:** 2021-06-07

**Authors:** Wenbin He, Meng Pang, Dung-Han Yeh, Jiapeng Huang, Philip. St. J. Russell

**Affiliations:** 1 Max Planck Institute for the Science of Light Staudtstrasse 2, 91058 Erlangen, Germany; 2 Department of Physics, Friedrich-Alexander-Universität, Staudtstrasse 2, 91058 Erlangen, Germany; 3 Present Address: State Key Laboratory of High Field Laser Physics, Shanghai Institute of Optics and Fine Mechanics, Chinese Academy of Sciences, Shanghai, 201800 China

**Keywords:** Solitons, Nonlinear optics, Fibre lasers, Mode-locked lasers

## Abstract

Mode-locked lasers have been widely used to explore interactions between optical solitons, including bound-soliton states that may be regarded as “photonic molecules”. Conventional mode-locked lasers normally, however, host at most only a few solitons, which means that stochastic behaviours involving large numbers of solitons cannot easily be studied under controlled experimental conditions. Here we report the use of an optoacoustically mode-locked fibre laser to create hundreds of temporal traps or “reactors” in parallel, within each of which multiple solitons can be isolated and controlled both globally and individually using all-optical methods. We achieve on-demand synthesis and dissociation of soliton molecules within these reactors, in this way unfolding a novel panorama of diverse dynamics in which the statistics of multi-soliton interactions can be studied. The results are of crucial importance in understanding dynamical soliton interactions and may motivate potential applications for all-optical control of ultrafast light fields in optical resonators.

## Abstract (web summary)

On-demand synthesis and dissociation of soliton molecules are controlled to occur simultaneously in massively parallel trapping potentials in an optomechanical lattice, unfolding the stochastic multi-soliton dynamics and enabling statistical insights.

## Graphical abstract

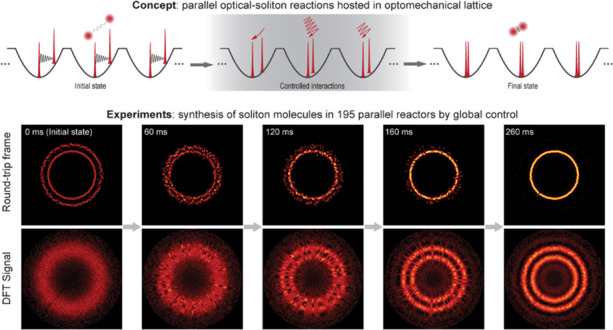

## Introduction

Temporal optical solitons in optical fibres, since first observed four decades ago^1^, have attracted widespread interest and stimulated foresight in applications that would potentially revolutionize optical communication^2^ and computations^3^ in the light of their particle-like properties. Naturally, interactions between solitons in fibres^4,5^ were brought under the spotlight as critical limitations or mechanisms in these applications, leading to a heap of progress since the 1980s. The studies on soliton interactions continue to date and are currently experiencing a vibrant renaissance, partly due to developments of the time-stretched dispersive Fourier transform (DFT) method^6^, which facilitates resolving of transient soliton dynamics, as well as due to trending focuses on soliton microresonators^7–9^, which as novel platforms, advance rapidly towards chip-scale integration. In parallel, many light−matter analogies have been suggested for multi-soliton complexes bound by soliton interactions^10–17^, epitomized by the “soliton molecules”^11,12^, which refers to closely bound solitons through direct interactions^18–21^. Reminiscent of real molecules synthesized from single atoms, optical soliton molecules behave like a single entity while displaying complex internal dynamics^12,13,22–25^, and have attracted considerable interest in both fundamental nonlinear physics and refreshed application promises such as ultrafast lasers^10,26,27^, spectroscopy^28^, optical communications^2,29^, and all-optical information processing^3,13,15,30^.

The light−matter analogy held by the soliton molecules can hardly, however, hide behind an obvious disparity: while real molecules usually participate in huge numbers in dynamic processes such as chemical reaction, soliton molecules have mostly been investigated as single entities. The cavity of a mode-locked laser, which has been routinely used as a platform for investigating complex soliton dynamics^10,12,22,31–34^, was conventionally able to host only few solitons generated out of random excitations^23,24^. These solitons are actually the result of dual balances, with gain and loss as dissipative factors in addition to Kerr-nonlinearity and dispersion hosted by the well-known “pure” solitons in conservative systems. These generalized solitons are usually referred to as dissipative solitons and are typically fixed-point attractors in nonlinear dissipative systems, in contrast to the “pure” solitons that usually feature a family of solutions in conservative systems^10^. The binding mechanism of soliton molecules could also be partially dissipative when formed in such nonequilibrium systems^23,32,35–38^ that incorporate spectra-filtering effects, nonlinear gain, saturation, etc., in addition to conservative interactions induced by cross-phase modulation (XPM). Previous studies mostly explored the soliton interaction from a limited number of experimental events collected randomly from each excitation, while to understand stochasticity of the complex soliton interactions from a higher level, large numbers of solitons and soliton complexes with controllable interactions are demanded, which, however, has long been a challenge in the experiments^13,15,16^. In particular, as topics that have recently aroused interest, the synthesis of soliton molecules from single solitons, and their dissociation into single solitons^10,39^, have not yet been experimentally demonstrated in a fully-controlled manner so far.

The physical scale of a mode-locked fibre laser is typically many times longer than an individual soliton, permitting in principle the coexistence of very large numbers of solitons and soliton molecules. In practice, however, uncontrollable drifting and collisions caused by noise^5,35,40,41^, together with intrinsic group velocity differences between solitonic elements^42–44^, have greatly limited the flexibility of mode-locked lasers as hosts for large solitonic structures. We have previously reported that the optomechanical lattice created in a mode-locked fibre laser^15^ by a short length of photonic crystal fibre^45^ (PCF) can synchronize the velocities of intra-cavity solitonic elements through long-range soliton interactions. Several solitonic elements can be temporally isolated and stably trapped within each time slot of the optomechanical lattice, forming a stable supramolecular structure^16^. In this work, we report that the consecutive time slots of the optomechanical lattice can function as parallel soliton “reactors” that can be controlled with all-optical techniques, echoing experiments on controlling chemical reactions by atomic manipulation^46^. By deliberately initiating the formation and dissociation of soliton molecules in these parallel reactors globally and individually using a variety of all-optical methods, we are able to uncover previously unexplored stochastic aspects of soliton−soliton interactions. The build-up of a stable soliton molecule, as observed in the experiments, generally requires multiple collisions^8,18,23,24^ before the eventual establishment of stable soliton−soliton spacing and phase. In contrast, the break-up of soliton molecules can occur in many different ways. We found that the motions of the solitons during these two processes followed a similar random-walk-like trajectory, accompanied by occasional radical repulsions and metastable states. While the statistical analysis on these processes revealed that the reaction rates of optical solitons followed simple mathematical relations, the first demonstration of fully controlled switching between different multi-soliton states in parallel optical-soliton reactors suggests many potential applications in information storage, data processing and logical operations using optical-soliton bits^13,15,29,30^.

## Results

### System configuration

**Fig. 1:**
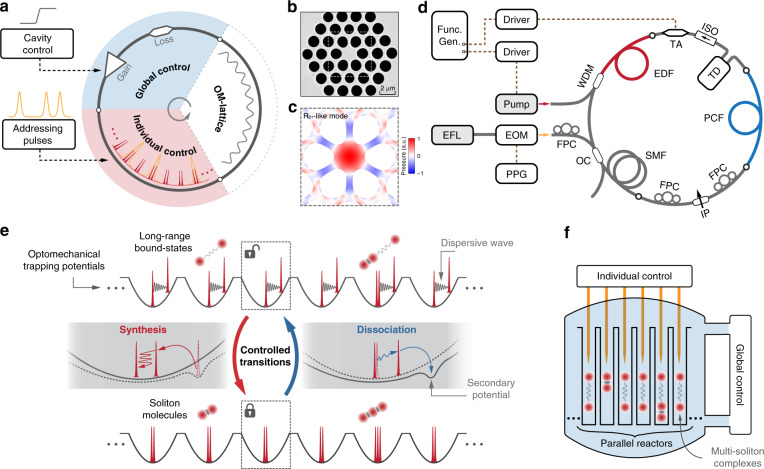
Conceptual illustration of parallel optical-soliton reactors in a fibre laser cavity. **a** Schematic of the parallel optical-soliton reactors based on a mode-locked ring-fibre laser cavity. The temporal optomechanical (OM) lattice provides trapping potentials to host parallel soliton interactions, while global and individual manipulations can be applied to control the interaction. **b** Scanning electron micrograph (SEM) of the PCF microstructure. **c** The *R*_01_-like acoustic mode in the PCF core simulated using the finite-element method. The displacement is exaggerated for clarity and the normalized pressure is indicated by the colour map. **d** Sketch of the experimental set-up, including an optoacoustically mode-locked fibre laser based on the PCF and external controllers. SMF single-mode fibre, EDF erbium-doped fibre, WDM wavelength-division multiplexer, FPC fibre polarization controller, TD tunable delay, TA tunable attenuator, IP in-line polarizer, OC optical coupler, ISO isolator. EFL erbium-fibre laser, EOM electro-optical modulator, and PPG programmable pattern generator. See Supplementary information (SI) Section I for more details. **e** Schematic of controlled soliton reactions in parallel trapping potentials. The solitonic elements trapped in each reactor can be transitioned between phase-uncorrelated long-range bound states and phase-locked soliton molecules, corresponding to the synthesis and dissociation of soliton molecules. **f** Artistic sketch of parallel reactors that host multi-soliton complexes as matter-like reactants, with global and individual control

The parallel optical-soliton reactors are realized using an optoacoustically mode-locked laser cavity in which a temporal optomechanical lattice accommodates a series of trapping potentials that can host parallel multi-soliton interactions^15,16^. The parallel reactors can be controlled either globally through cavity parameters (e.g., laser gain and cavity loss) or individually through perturbations from external addressing pulses (see Fig. 1a). In the experiments, we use a 2-m-long solid-core PCF with a core diameter of 1.9 µm to provide an *R*_01_-like mechanical resonance^45^ at 1.887 GHz (see Fig. 1b, c). We implemented the PCF in a conventional mode-locked erbium-fibre laser cavity such that the optically driven acoustic vibration in the PCF core could divide the ~20-m cavity (~104-ns round-trip time) into 195 time slots, forming a self-organized and self-stabilized optomechanical lattice^15^ (see the laser set-up in Fig. 1d). This temporal lattice was adjusted to host a variety of solitonic supramolecules^16^ at pump powers above 600 mW. In such structures, each time slot of the optomechanical lattice, functioning as a potential well, can stably trap one or more solitons bound by long-range and/or short-range forces (see Fig. 1e), while the entire structure can accommodate a large population of solitons and soliton molecules, all sharing the same group velocity.

The long-range bound, phase-uncorrelated soliton states^16^ are ideal initial/terminal states for the synthesis/dissociation of phase-locked soliton molecules. The long-range bound state results from the balance between the repulsive force exerted by the dispersive waves and the attractive force exerted by the acoustic waves. In order to initiate soliton reactions, the repulsive force needs to be controlled. An abrupt decrease of long-range repulsive forces within the traps can cause the solitons to move towards each other, resulting in multiple collisions and eventually the formation of a soliton molecule. Conversely, soliton molecules can be broken up by enhancing the repulsive forces, causing the solitons to dissociate and settle down into phase-uncorrelated long-range bound states (Fig. 1e). In experiments, the reactions between solitons can be initiated by perturbing specific laser parameters that affect the solitons in all trapping potentials of the lattice, as the global-control method. In this case, we can choose to perturb the laser gain by changing the driving current of the pump diode laser, or to perturb the cavity loss by using a fast tunable attenuator based on Pockels effect (see Fig. 1d). Reactions can be initiated also in selected trapping potentials by launching addressing pulses into the laser cavity to perturb the soliton interactions through cross-phase modulation^15^ (XPM), i.e., the individual control. The addressing pulses can be produced by electro-optically modulating a continuous-wave laser using a programmable pattern generator with a synchronized time grid (see Fig. 1d). The individual control allows each solitonic element to be reversibly and selectively edited (see “Results” below) without affecting others. The parallel trapping potentials that can host controllable reactions between solitonic elements are reminiscent of the chemical reactors, as conceptually sketched in Fig. 1f, although the “reactants” are optical solitons and their complexes, instead of real atoms and molecules.

### Synthesis of parallel soliton molecules

**Fig. 2:**
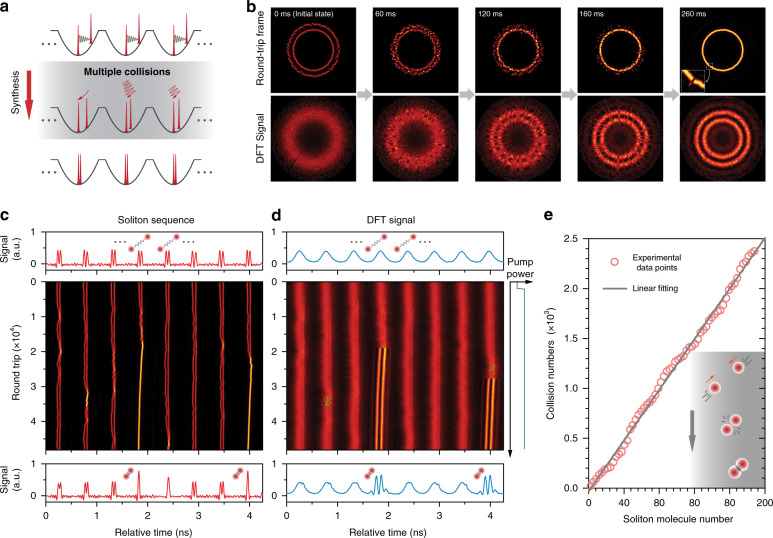
Global synthesis of phase-locked soliton molecules. **a** Schematic of the synthesis of soliton molecules in parallel reactors. In each potential, two solitons are prepared in a long-range phase-uncorrelated bound state. After an abrupt change in the pump power or the cavity loss, the long-range binding collapses and the two solitons experience multiple collisions before forming a stable soliton molecule. **b** Top panels: selected frames from an experimental recording of the synthesis process over all 195 reactors, plotted in cylindrical coordinates (see “Methods”). Bottom panels: the corresponding DFT signal. The initial state contains 194 time slots with two long-range bound solitons and a reference time slot with a single soliton (see zoom-in inset in the last upper-row panel). The gradual establishment of spectral fringes in the DFT signal over all time slots indicates the formation of phase-locked soliton molecules. See Supplementary Movie 1 for the complete recording. **c** Time-domain evolution in eight consecutive time slots over the initial 49,000 round trips (~5 ms). **d** The corresponding DFT signal. Soliton molecules are formed in two time slots, as indicated by the stable fringes in (**d**). Top and bottom panels in (**c**) and (**d**) show the recorded signal for the initial and final round trips. **e** Cumulative number of soliton collisions is proportional to the number of soliton molecules in all 195 reactors during a single synthesis (red circles). The grey line is a linear fit.

To investigate the dynamics of soliton-molecule formation, we prepared a stable soliton supramolecule as the initial state (as sketched in Fig. 2a), consisting of 195 time slots, all with two long-range bound solitons, except for a reference slot containing a single soliton (see Fig. 2b and “Methods”). In the absence of repulsive forces, the solitons within a single time slot will tend to collide. This is prevented in practice by the repulsive forces that arise from the shedding of dispersive waves^5,47,48^, leading to the formation of a secondary trapping potential (Fig. 1d) that causes long-range binding at ~60-ps separation (Fig. 2b, c)—much longer than the ~1-ps duration of the solitons (see SI Section II) and can be continuously tuned over a wide range. As a result, the two trapped solitons have negligible field overlap and are thus uncorrelated in phase^5,16^, which has also been experimentally verified using the heterodyne measurement in our previous experiments (see Supplementary Fig. 7 of ref. ^16^).

Synthesis was initiated by abruptly changing the pump power or the cavity loss, which causes a decrease in the inter-soliton repulsive force (see SI Section II) and a gradual reduction in soliton spacing in all the reactors, culminating in multiple soliton collisions (Fig. 2a). We studied the real-time motion of the reacting solitons by time-domain recording (limited by the bandwidth of the photodiode, so suitable only for long-range binding) and DFT signal (suitable for short-range (<14 ps) binding) in all the reactors (see “Methods”). The entire reaction process over all time slots, initiated by pump perturbation, was first recorded at a 5-kHz frame rate and is shown in Fig. 2b in cylindrical coordinates for five selected frames (see Supplementary Movie 1 and SI Section III). The recordings over the initial 49,000 round-trips (~5 ms) in eight consecutive reactors (out of 195) are plotted in Fig. 2c, d, showing the soliton dynamics on finer time scales.

The experimental results indicate that the formation of a stable soliton pair, resembling a molecule, generally requires multiple collisions within the reactors before an effective collision that establishes a stable spacing/phase relation between the solitons. As shown in Fig. 2c and Supplementary Movie 1, while in some reactors a stable soliton pair formed within 5 ms after only a few soliton collisions, in many other reactors, tens to hundreds of collisions were required. On average, an effective collision occurred out of ~13 collisions, as we estimate from the recording. To examine the statistics of the synthesis, the cumulative collision numbers in all the reactors are plotted in Fig. 2e against the total number of soliton molecules, measured at time intervals of 50 µs. The result exhibits approximately a linear dependence, indicating that the rate of soliton molecular formation is proportional to the rate of soliton collisions, even though that these rates themselves were observed to vary over the reaction time (see SI Section IV). The result can probably be interpreted as a consequence of phase-sensitive interaction between the two solitons. Given that the two solitons in each reactor have uncorrelated phase evolution before a collision, the formation of a soliton molecule can only occur as a probabilistic event with a fixed probability out of each collision, while the trapping potential ensured the everlasting occurrence of such collisions until an effective one that brings the solitons into a bound state.

Once formed, the soliton molecules would propagate as single entities with precisely synchronized phase and group velocities between the bound solitons. However, they would generally differ from single solitons in group velocity^42–44^, and as a consequence, gradually shift to slightly different positions within their time slots, while remaining trapped by the optoacoustic trapping potential, as seen in Fig. 2c, see also the reference slot in the final state in Fig. 2b. The optomechanical lattice was robust enough to host all the soliton reactions until they are completed, without destabilization.

**Fig. 3:**
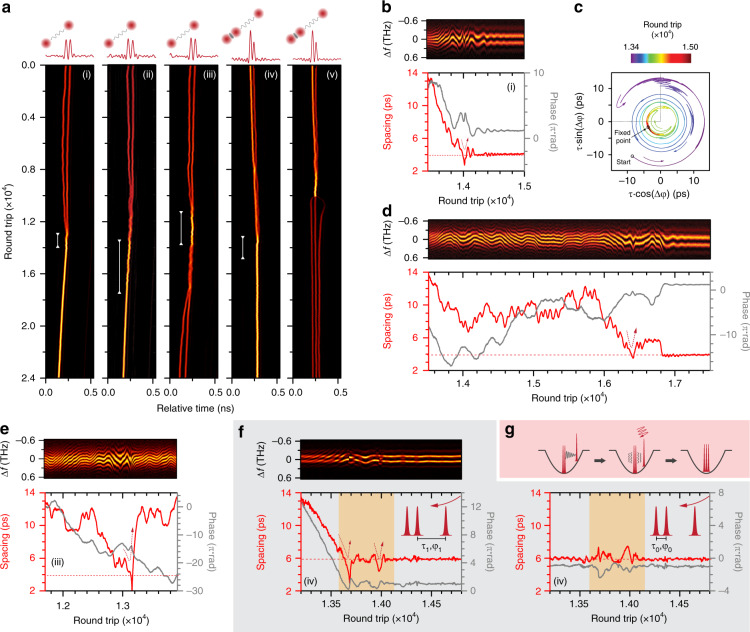
Dynamics of soliton-molecule synthesis. **a** Panels (i)−(iii) time-domain recordings in three selected time slots showing reactions between long-range bound solitons (~60-ps spacing) and soliton molecules (~3.8-ps inner spacing). Panels (i) and (ii) show successful synthesis, while panel (iii) shows dissociation after a collision. Panels (iv) and (v) show reactions between a single soliton and soliton-pair molecule (~50-ps inner spacing), resulting in the formation of a soliton triplet (~6-ps inner spacing, panel (iv)), and in repulsion between all three solitons (panel (v)). **b−e** DFT signal (upper) and retrieved spacing and phase evolution (lower) for the ranges marked by white lines in panels (i)−(iii) (**c** is the interaction-plane plot of **b**). The horizontal dashed lines mark the spacing of a stable molecule. **f** DFT signal (top panel) and the retrieved spacing and phase relation between the neighbouring solitons (lower panels) during soliton-triplet formation (panel (iv) in **a**). The horizontal dashed lines mark the minimum soliton spacing (~6 ps) in the final state. **g** Schematic of soliton-triplet formation. A single soliton collides with a soliton pair multiple times, disturbing the original molecular bond and resulting in the formation of a soliton triplet

The stochasticity of soliton-molecule formation can be revealed from the transient multi-soliton dynamics after the initiation of the reactions over the hundreds of parallel soliton reactors. In practice, we can retrieve the states of the multi-soliton structure in each time slot using the corresponding DFT signal in which the period and offset of the interferometric fringes indicate the instantaneous soliton spacing and relative phases^12^ (see “Methods” and SI Section II). Panels (i)−(iii) in Fig. 3a (with the corresponding trajectories in Fig. 3b−e) show the reaction processes in three parallel reactors in which the two solitons in each time slot attempt to transit from a phase-uncorrelated long-range bound state (~60-ps spacing) to a phase-locked soliton molecule (3.8-ps spacing and π-phase difference) after the pump power was perturbed (same as in Fig. 2). The π-phase soliton molecule results from the balanced interaction between repulsions induced by XPM and attractions induced by spectral filtering^21^. We found using numerical simulations that the gain-filtering effect imposed by the Er-doped gain section can cooperate with the XPM effect to result in a stable soliton molecule with a phase difference of π and a fixed spacing (see “Methods” and SI Section VI), while in panel (i) the formation process is completed within 1000 round trips following a rather simple trajectory (spacing and phase evolution in Fig. 3b and interaction plane^20^ in Fig. 3c); the reaction shown in panel (ii) lasted more than 3000 cavity round-trips and followed a more complex trajectory (see Fig. 3d). Panels (i) and (ii) show how effective collisions could take place in a reactor, while panel (iii) shows a more frequently observed case of a soliton collision that lasts thousands of cavity round trips without, however, giving birth to a soliton molecule (see Fig. 3e). Instead, the two solitons strongly repel each other, before drifting towards the next collision.

We observed that the soliton motion at most separations was stochastic, reminiscent of a one-dimensional random walk with fixed step length^49^. This is probably caused by phase-dependent inter-soliton forces^4,18^ that are constantly varying in strength and flipping in direction. Non-solitonic components such as dispersive waves^48^ could also participate in the soliton interaction event during their evolution in the mode-locked cavity. We observed similar random-walk-like motion of the interacting solitons in numerical simulation (see SI Section VI). In addition, the initial phase difference between two solitons before binding is also random^16^, probably accounting for the widely different trajectories from reactor to reactor. When the soliton separation is less than the molecular spacing (<3.8 ps), however, a strong repulsive force emerges^4^, which quickly pushes the solitons apart. This is a ubiquitous feature not only in the synthesis of soliton molecules but also in their dissociation, as described below (see SI Section V). In our numerical simulation, we also observed radical soliton repulsions as a typical dynamical feature during the soliton interactions (see SI Section VI). We suspect that the radical repulsion is mainly due to dissipative factors (gain saturation, peak-power clamping, etc.), which prevent the two solitons to merge into one with a higher peak power. Only in effective collisions, the colliding solitons would enter a converging trajectory with quickly damped spacing and phase relation (see DFT signal in Fig. 3b, d).

The synthesis between different solitonic elements has also been realized in a controlled manner using the parallel reactors, which was previously challenging due to their intrinsic velocity discrepancy that causes uncontrolled collisions. In the experiment, we first prepared a soliton supramolecule in which most time slots hosted a single soliton bound with a soliton-pair molecule, their group velocities being synchronized by long-range interactions^16^ such that they would not collide freely before the initiation^50^. Then we abruptly increased the intra-cavity loss using the fast tunable attenuator, which weakened the dispersive-wave perturbation (see SI Section II). Consequently, attraction overcame repulsion, initiating the soliton reactions. Two examples of three-soliton reactions are shown in panels (iv) and (v) of Fig. 3a. In panel (iv), collisions between the soliton pair and the single soliton resulted in the formation of a phase-locked soliton triplet. The measured trajectories between neighbouring solitons during synthesis in panel (iv) are shown in Fig. 3f. The single soliton collides strongly with the soliton pair, resulting in a strong disturbance to the soliton pair (highlighted in yellow in Fig. 3f) before the establishment of a second molecular bond (the reaction process is sketched in Fig. 3g). In panel (v), however, a similar collision results in dissociation of the soliton pair molecule, followed by a strong repulsion between all three solitons, highlighting the complexity during the three-soliton reactions (see SI Section VI).

### Dissociation of parallel soliton molecules

**Fig. 4:**
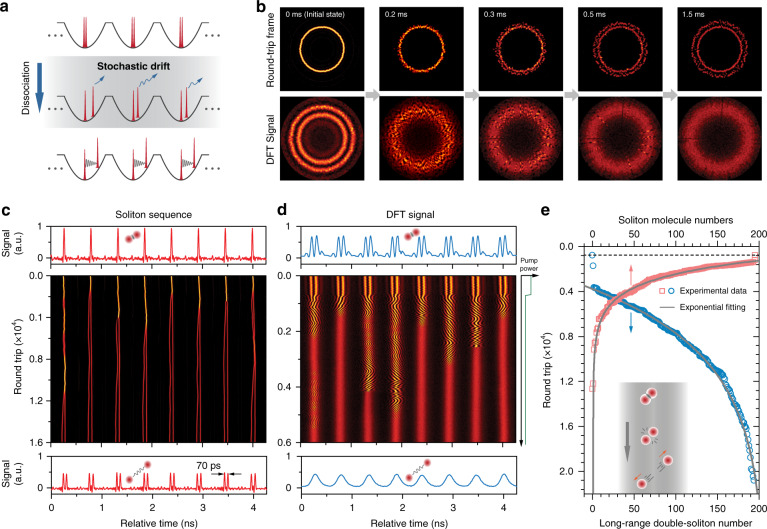
Dissociation of soliton molecules in parallel reactors. **a** Schematic of the dissociation process in the case when all the reactors contain identical soliton molecules. By perturbing the pump power or the cavity loss, the molecular bonds collapse and the solitons start to drift stochastically and diverge, before reaching a stable long-range bound state. **b** Top panels: selected frames from experimental measurements of the dissociation process in all 195 reactors, plotted in cylindrical coordinates. Bottom panels: the corresponding DFT signal. Initially, all the time slots host identical soliton molecules with a spacing of ~3.8 ps and a relative phase of ~π. The gradual smearing out of interferometric fringes in the DFT signal indicates dissociation. See Supplementary Movie 2 for the complete recording. **c**, **d** Time-domain sequence in eight consecutive time slots over the first 16,000 round trips (~1.8 ms) and the corresponding DFT signal, exhibiting instant collapse of the molecular bonds following pump-power perturbation. The dissociation trajectories are highly diverse, as indicated by fringes in (**d**). Top and bottom panels in (**c**) and (**d**) show the signal traces for the initial and final round trip. **e** Populations of soliton molecules (red squares) and long-range double solitons (blue circles) over all 195 reactors, plotted against round trip number during dissociation, fitted to exponential functions (grey curves). The horizontal dashed line marks the perturbation time

Phase-locked soliton molecules can also dissociate into uncorrelated single solitons under global control (Fig. 4a). A typical example is recorded and plotted in cylindrical coordinates in Fig. 4b for five selected frames (for full recording see Supplementary Movie 2). The reaction is initiated by a slight decrease in pump power, which causes the repulsion induced by dispersive waves to overcome the attraction and therefore, the rapid break-up of the soliton molecules. This process is much faster than the soliton-molecule synthesis that generally requires multiple collisions. The dissociation follows highly diverse trajectories from reactor to reactor, as seen in Fig. 4c (time domain) and Fig. 4d (DFT). We attribute the stochastic fluctuations during the early stages of dissociation to noise-like repulsive forces between the solitons exerted by randomly excited dispersive waves^5,47^ along with other inevitable noises in the cavity. After dissociation, long-range binding between the solitons was gradually established, eventually settling down after a few milliseconds.

Since soliton molecules dissociate over diverse trajectories, the dissociation rate can only be determined statistically. We first define criteria for determining the completion of a reaction: full dissociation for a separation of 14 ps or greater (the maximum spacing retrieved from the DFT signal), and long-range soliton binding for a separation of 55 ps or greater. We plot the total number of soliton molecules and long-range double solitons during dissociation against the number of round trips (Fig. 4e). Both measurements are roughly exponential, indicating that the dissociation rate is proportional to the instantaneous number of undissociated reactants. This allows us to estimate a soliton-molecule “half-life” of ~1200 round trips (~120 µs) using the 14-ps criterion (see SI Section IV for details).

**Fig. 5:**
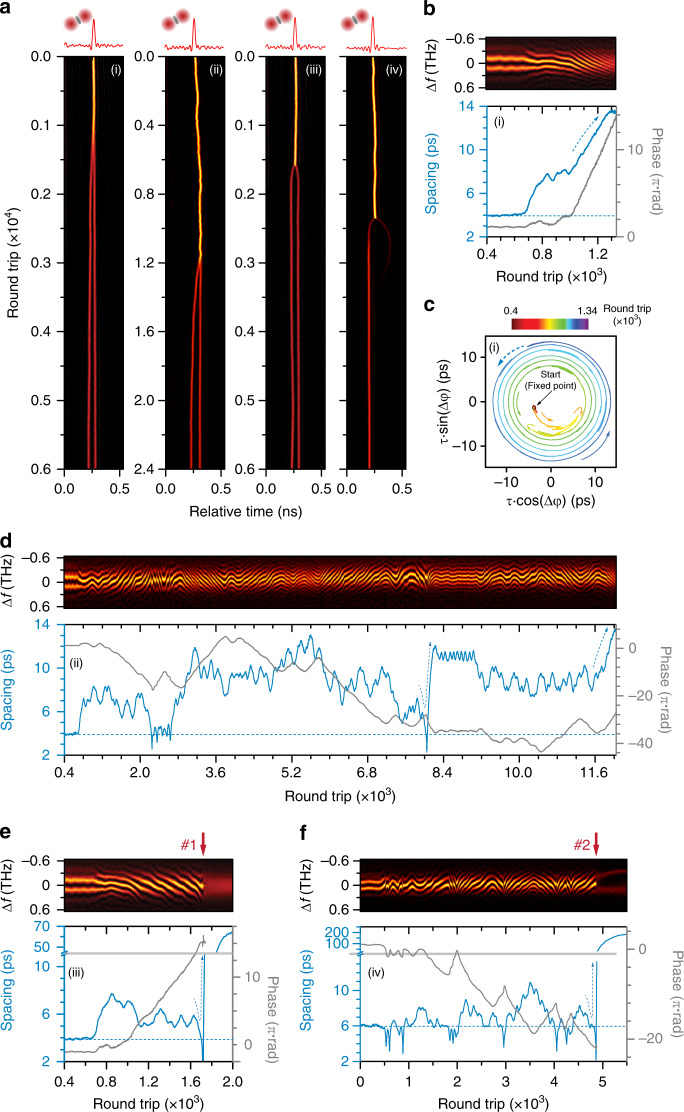
Dissociation dynamics of soliton-pair molecules. **a** Time-domain recordings of soliton-molecule dissociation in four selected reactors. Panel (i) shows a rapid dissociation with a rather smooth trajectory as seen in the DFT signal in (**b**) and plotted in the interaction plane in (**c**). Panel (ii) shows a rather longer dissociation process in a different reactor, with a random-walk-like trajectory as shown in (**d**). Panel (iii) shows a dissociation that ended with the abrupt repulsion of the two solitons after the spacing falls below ~ 3.8 ps, as retrieved from the DFT signal shown in (**e**). Panel (iv) shows a dissociation that causes one soliton to be eliminated due to the abrupt repulsion (see DFT signal in (**f**)). See details of the abrupt repulsion (regions marked by red arrows in (**e**) and (**f**)) in SI Section V

The soliton motion during dissociation retrieved from the DFT signal, was found to share many characteristics with that of synthesis. Figure 5a shows a few dissociation trajectories recorded in the parallel reactors initiated by perturbing the pump power. Panel (i) shows fast dissociation (<1000 round trips) with a relatively smooth trajectory, as indicated by the retrieved spacing and phase (Fig. 5b, c). Panel (ii) shows another trajectory recorded within the parallel reactors, which, however, exhibit a rather long dissociation lasting >10000 round trips with a random-walk-like trajectory (see Fig. 5e). Within the trajectory, we can notice a “metastable state” at a spacing of ~11 ps at which the two solitons temporally reside with quasi-stable relative phases (~π), which might correspond to a fixed-point attractor with low stability. Similar phenomena also appeared in other reactors. In addition, we can notice within the trajectory that a strong repulsion^4^ occurred (indicated by the blue dashed arrow) when solitons temporally reached a spacing below the initial value (marked by the dashed horizontal line) during the random walk. This phenomenon was also observed in synthesis dynamics (e.g., Figure 3e) and was found to be universal during the reactions (see SI Section V). These features can also be fully reproduced in our numerical simulations (see SI Section VI), while the parallel reactors unfolded them efficiently in a unique panorama of soliton dynamics.

In a few reactors, such inter-soliton repulsion can be so radical that the dissociation can terminate immediately. One example is shown in panel (iii) of Fig. 5a (with DFT signal in Fig. 5e), in which the abrupt drop in soliton spacing triggered strong repulsion between the solitons within only a few round trips, followed by the quick establishment of long-range binding. Occasionally, one (or both) of the interacting solitons was extinguished after such radical repulsion, as shown by the example in panel (iv) of Fig. 5a (with DFT signal in Fig. 5f). This is probably due to the fact that the carrier frequencies of both solitons are significantly shifted during the radical repulsion, which affects their gain/loss balance in the laser cavity with a limited gain bandwidth. The diverging soliton then experiences net loss and failed to recover before the long-range forces (trapping potential) shift the frequency back (see SI Section V for more details).

**Fig. 6:**
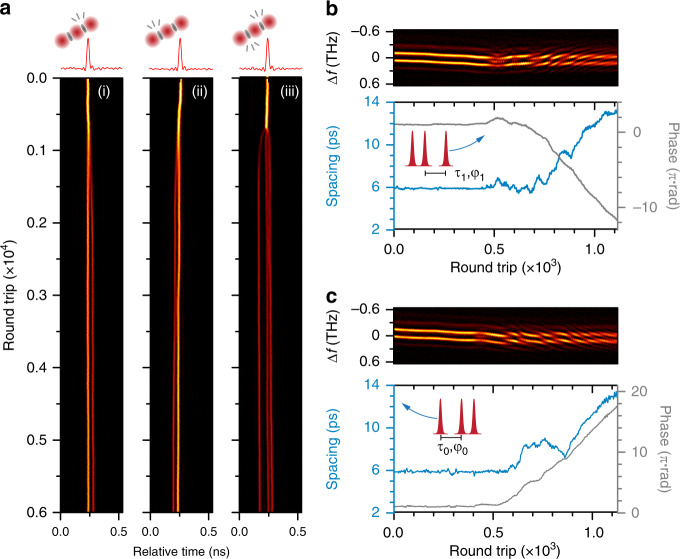
Dissociation dynamics of soliton-triplet molecules. **a** Time-domain recordings of soliton-triplet dissociation in three selected reactors. In panels (i) and (ii), the dissociation breaks either of the two molecular bonds, resulting in a soliton-pair molecule and a single soliton with different orders. The corresponding DFT signal is shown in (**b**) and (**c**). In panel (iii), both molecular bonds are severed, resulting in three uncorrelated solitons

Dissociation of soliton triplets follows even more complex dynamics, as seen when the system is loaded with soliton triplets in each reactor and then perturbed by decreasing the cavity loss. Three examples recorded within the parallel reactors are shown in Fig. 6. Panels (i) and (ii) show dissociations that broke either of the two molecular bonds within the triplet, leading to different orientations between the soliton pair and the single soliton in the final long-range bound state (see DFT signal in Fig. 6b, c). In panel (iii), both molecular bonds between the three solitons are severed, resulting in three phase-uncorrelated single solitons. see SI Section VII for more examples). The retrieval of soliton spacing and phase in three-soliton interaction is less intuitive than in the two-soliton case, and currently, we can only resolve interactions that do not involve radical collisions due to phase and spacing ambiguities (see SI Section II). Recent studies have demonstrated the possibility to use machine-learning strategies^51^ for resolving complex multi-soliton dynamics without ambiguities. The unique parallel-reactor scheme demonstrated here may provide an ideal platform for massive training and implementation of various machine-learning algorithms.

### All-optical control of soliton reactions in selective reactors

**Fig. 7:**
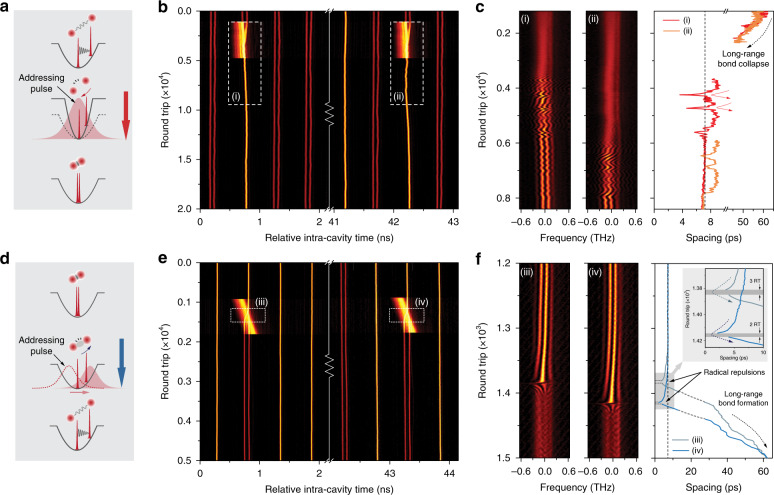
Control of reactions in selected time slots by addressing pulses. **a** An external addressing pulse overlaps continuously with an initially prepared long-range soliton pair, causing strong attractive forces between them that result in the formation of a soliton molecule. **b** Time-domain recordings of two processes in reactors hosting long-range soliton pairs. A 200-ps-long addressing pulse overlaps with the solitons over ~3000 round trips, causing the two solitons to move closer, interact and form a soliton molecule. **c** DFT signal marked by the white dashed boxes (i) and (ii) in (**b**), together with the evolution of the inter-soliton spacing. **d** Schematic of soliton-molecule dissociation by an external addressing pulse. The addressing pulses traverse the soliton molecule, quickly severing the molecular bond. The two dissociated solitons become long-range bound in the end. **e** Time-domain recordings of the dissociation processes in reactors containing soliton pairs. Addressing pulses with a slight repetition-rate offset “walk through” of the soliton molecules over 200 round trips, causing breakage of the soliton molecule, followed by the establishment of the long-range binding. **f** DFT signal marked by the white dashed boxes (iii) and (iv) in (**e**), together with the evolution of the inter-soliton spacing. Strong repulsion can be observed when the spacing falls below ~5 ps, which occurred within 2−3 round trips, as shown in the inset

Soliton interactions within selected reactors can be controlled by launching a sequence of precisely timed optical pulses into the laser cavity (Fig. 1d)^13,15,30^, permitting individual solitonic elements to be edited by XPM (see “Methods” and SI Section VII). To demonstrate this, we first prepared a soliton supramolecule in which a mixture of long-range double-soliton and phase-locked soliton pairs exist in the time slots. To convert two long-range bound solitons into a soliton molecule in a selected time slot, we launched a train of ~200-ps pulses at the cavity round-trip frequency, precisely timed to interact with targeted time slots over ~3000 round trips (see Fig. 7a, b). Since the two solitons initially ride on different amplitude upon each addressing pulse, the two solitons would see different XPM-induced nonlinear index and tend to move closer. Therefore, an effective “attraction force” was applied to the solitons that exceeded their long-range repulsion, leading to soliton collisions and formation of stable soliton molecules (as shown in DFT signal and retrieved trajectory in Fig. 7c from the selected time slots). The addressing pulses are generated using a PPG, following a time-grid, which equals that of the cavity laser. Therefore, the selection of time slots to be perturbed is directly achieved in the pattern editing in the PPG, while repetitive launching of the addressing pulse pattern leads to the accumulation of perturbations in the selected time slots^15^. Typically, the addressing pulse pattern needs to be launched a few thousand times so as to repetitively “hit” the targeted time slots to ensure sufficient overlapping time with the solitons. In practice, the addressing pulses only co-propagate with targeted intra-cavity solitons over a several-metre-long SMF section before getting eliminated by the intra-cavity polarizer to prevent laser gain depletion. Pre-adjustment of the optical polarization state using the FPCs needs to be implemented before initiating the soliton reactions (see SI Section VIII for details).

Conversely, to break apart soliton molecules in selected reactors, we developed a special trick that made use of the same train of addressing pulses as above with however a slight offset in repetition rate. In this process, the addressing pulses would effectively “walk through” the soliton molecule, flipping the phase relation of the soliton molecule from π to 0, causing strong attraction and thus compressed spacing (Fig. 7d). The compressed soliton spacing then led to radical repulsion that pushed the solitons apart, i.e., dissociated the soliton molecule (similar to the case in Fig. 5e). The addressing pulses in this case operate like optical “scissors”, severing the molecular bond while traversing it. By a suitable choice of repetition-rate offset (empirically set as *f*_ext_ – *f*_cav_ ≈ 20 Hz, see Fig. 7e), we achieved deterministic dissociation of selected soliton molecules. The trajectories retrieved from the DFT signal are shown in Fig. 7f, revealing that the traversing pulse first compressed the soliton separation to below ~5 ps, triggering strong repulsion within 2–3 round trips, and eventually leading to the collapse of the molecular bond and establishment of the long-range binding.

The initial phase-relation shift (π to 0) imposed by the traversing pulse is probably induced by a mutual frequency shift of the interacting solitons. We suspect that the nonlinear polarization rotation (NPR) of the targeted soliton molecule was gradually modified^15^ during the effective “walk-through” of the addressing pulses, leading to a varying loss at the polarizer, which was then translated into a carrier-frequency shift by laser gain-filtering effect. In addition, the soliton-dragging effect^52^, as a pure nonlinear process, might also contribute to the frequency shift of the soliton molecule as the addressing pulses induced a varying nonlinear index during the walk-through. Once the soliton spacing was strongly compressed, the phase relation instantly flipped back (0 to π), probably due to an additional, self-induced frequency shift, which turned the force direction back into repulsion (similar to the case shown in Fig. 5e which though occurred in the absence of addressing pulses). See SI Section VIII for details.

The individual control we illustrated here, provides enhanced flexibility upon the parallel-reactor control scheme, which would be potentially useful when combined with global control in fast and precise manipulation of a massive number of solitons, which is essential in applications like soliton communications and soliton-based optical computations. Nevertheless, the exact control mechanism also requires further explorations and optimizations to achieve more efficient and on-demand flipping of multi-soliton states, particularly when more complex structures are hosted in the reactors.

## Discussion

The parallel optical-soliton reactors formed in a mode-locked fibre, constitute a unique experimental arena for multi-soliton dynamics, boasting highly controllable soliton interactions as well as significant scaling of the soliton population. The prominent controllability stems from the regular and robust confinement in each optomechanical lattice cell together with various methods for tailoring soliton interactions, namely the global control through cavity parameter adjustment and the individual editing through addressing pulses. The scaling of the soliton population, on the other hand, benefits from the geometric advantage of fibre-loop configuration. As a trade-off, the parallel reactors pre-group the hundreds of solitons in regular time slots to avoid uncontrolled pulse bunching^53,54^ that may lead to system collapse without, meanwhile, complete isolation between the solitonic elements that deprive the flexibilities of this platform. As a result, we can realize “active” observations though controlled initiation, which can efficiently unfold the diversity of multi-soliton dynamics through the massively parallel reactions and moreover reveal collective patterns concealed under the stochastic appearances of individual events.

The synthesis and dissociation of soliton molecules, as highly interested dynamic processes for establishing the light−matter analogy, are realized on-demand for the first time using the parallel-reactor scheme and result in a series of observations with enriched physical insight. We revealed that the interacting solitons followed a random-walk-like motion while residing occasionally on some “metastable states”. Moreover, strong soliton repulsions occurred whenever the soliton spacings reached below the molecular spacing, which can lead to fast dissociation of soliton molecules. Despite the stochasticity of each individual reaction, simple statistical rules were found out of surprise that govern soliton reactions at a collective level. Multiple collisions were found to be essential for soliton molecule synthesis, while the rate of molecule formation depends linearly on the rate of soliton collisions over all reactors. Such statistical rules are actually reminiscent of the collision theory of chemical kinetics, which states that the rate of a gas-phase reaction is proportional to the collision frequency^55^. The rate of soliton-molecule dissociation, on the other hand, was found to be proportional to the instantaneous population of undissociated soliton molecules, which also remind us of the first-order reaction model in classical chemical kinetics^55^.

With the capability of hosting massive interaction events simultaneously, the parallel reactor system offers an opportunity to explore the various light−matter analogies of solitons (including soliton molecules, soliton rains, soliton crystal, etc.) at a collective level, in contrast to previous studies that plateaued at the single-entity level. As well as permitting a higher-level exploration of soliton dynamics, the parallel-reactor scheme provides a platform for testing new methods for controlling of soliton interactions. By further scaling of soliton population (with e.g., longer-cavity and wider trapping potentials) and optimization of the control method, the parallel-reactor scheme may become qualified to serve a practical purpose in all-optical information processing that massively uses solitons as data bits, e.g., all-optical logic gate^3^, non-binary bit storage^15,30^ and transmission^2,29^.

A series of other possibilities can also be explored using parallel optical-soliton reactors. Firstly, light−matter analogies placed upon many multi-soliton states can be further enriched in various aspects, e.g., by inspecting energy changes during soliton reactions^56^ and comparing with that in chemical reactions. Secondly, other than the soliton molecule dynamics, this system could also be used to re-examine phenomena such as soliton explosions^57^, pulsation^34,58^, fragmentation^23,58^, resonant vibrations^13,33^ as well as the build-up^26^ and extinction^24^ of solitons from a collective viewpoint. The isolation between all the parallel reactors could also be possibly manipulated in the following development to allow weak interactions between the reactors, making this platform suitable for studying potential mutual synchronization effects^59^ in multi-soliton dynamics.

The parallel-reactor scheme may also be realized using a different mechanism, e.g., with trapping potentials driven by external light^30^ or an intra-cavity modulation controlled by an external signal, so as to enhance the controllability. Other platforms, especially the microresonators, may also see the potential to implement a similar concept of “parallel-reactor”. Although an individual microresonator can hardly host a large amount of optical solitons or to utilize their long-range interactions as in fibre laser, a massive array of microresonators can be easily integrated and highly controlled on a chip-scale platform, which probably has the potential to unfold a similar panorama of soliton dynamics.

## Materials and methods

### Preparing soliton supramolecule states

In order to generate the desired soliton supramolecule state, we need to choose a proper working point for the laser, adjusting the pump power level, cavity loss and most importantly, carefully aligning the intra-cavity FPCs to set a proper bias for NPR. Dispersion-compensating fibres (DCFs) are also used in the cavity to properly tailor the dispersive waves. The net cavity dispersion for the mode-locked cavity under global control is calculated to be −22.2 ps^2^ km^−1^, which leads to the conventional soliton-regime operation of the fibre laser. In the individual control experiment, an additional 10-m-long SMF is added to the cavity in order to enhance the XPM effect between the addressing pulses and the intracavity soliton, which leads to slightly a higher dispersion of −22.3 ps^2^ km^−1^. In addition to the fast tunable optical attenuator, a manual tunable attenuator was also inserted into the cavity to introduce a loss bias—crucial for finding a proper working point for the parallel reactions.

### Numerical simulation of soliton molecules

We built up a Python-based numerical simulation model for investigating the multi-soliton dynamics in a mode-locked fibre laser cavity. In each fibre section, the model employs a scalar nonlinear Schrödinger equation (NLSE) and performs the calculation using symmetrized split-step Fourier transform method. Other components are considered as lumped elements in the cavity (e.g., optical couplers, the saturable absorber and the tunable attenuator). The generalized scalar NLSE we considered for each fibre sections is

$$\frac{{\partial A}}{{\partial z}} = i\mathop {\sum}\limits_{n = 2}^\infty {\frac{{i^n\beta _n}}{{n!}}\frac{{\partial ^nA}}{{\partial t^n}}} + {\mathrm{i}}\gamma |A|^2A + \frac{g}{2}A + \frac{g}{{2\Omega _g^2}}\frac{{\partial A}}{{\partial t^2}}$$

where *z* is the propagation distance, *t* is the relative time in the moving frame and *A*(*z*, *t*) is the field envelop of the multi-solitons so normalized that its square is directly the instantaneous power in the optical fibre. The first term on the right-hand side (RHS) describes the spectral phase accumulation induced by fibre dispersion of all orders. In our experiments, the pulse duration is ~1 ps; therefore, we only consider dispersion up to third-order (*β*_2_ and *β*_3_) in this numerical simulation. The second term denotes the SPM effect in the optical fibre with nonlinear parameter *γ*. For SMF, *β*_2_ = −22.5 ps^2^ km^−1^, *β*_3_ = +0.12 ps^3^ km^−1^, *γ* = 1.3 km^−1^ W^−1^. For PCF, *β*_2_ = −157 ps^2^ km^−1^, *β*_3_ ≈ +0.1 ps^3^ km^−1^, *γ* = 33 km^−1^ W^−1^. For EDF, *β*_2_ = +77 ps^2^ km^−1^, *γ* = 9.3 km^−1^ W^−1^. We also introduced a short piece of DCF in the cavity to finely adjust the Kelly sideband. For DCF, *β*_2_ = +121 ps^2^ km^−1^, *γ* = 3.4 km^−1^ W^−1^. The third term describes the gain/loss in the optical fibre (gain applicable in the EDF). The saturable gain coefficient *g* is defined as $g = g_0\exp \left( { - {\int} {|A|^2{\mathrm{d}}t/E_{{\mathrm{sat}}}} } \right)$where *g*_0_ is the small-signal *g*ain coefficient, and *E*_sat_ is the saturation energy of the gain fibre. The gain-filtering effect in EDF is described by the fourth term with an approximated parabolic profile (bandwidth of Ω_g_.) The artificial saturable absorber induced by the NPR effect is simplified into a transmission function of $f(t) = 1 - l_0\exp \left( { - |A(t)|^2P_{{\mathrm{sat}}}} \right)$ where *l*_0_ is the low-intensity loss and *P*_sat_ is the effective saturation power. The temporal window used in our simulation is 512 ps digitized with two^13^ data points. The intrinsic losses of all fibre sections are negligible, while a few lumped losses are inserted into the simulation model in accordance with the real experiment set-up. For the synthesis simulation, we also induced an effective index-trapping potential (with a modulation depth of 10^−7^) in order to induce soliton collisions, which is induced by acoustic vibrations in real experiments. Meanwhile, the stability of the soliton molecule, as we found using the simulation model, is irrelevant to the presence of the index-trapping potential. To simulate the dissociation process, we set the stable soliton molecule out of the synthesis process as the input, and then induce a slight perturbation upon the gain parameter. See SI Section VI for the detailed settings and results of the numerical simulation.

### Soliton sequences plotted in cylindrical coordinates

The soliton sequence propagating in the laser ring cavity follows a regular time grid, each time slot hosting one or more solitons. To simultaneously illustrate the soliton dynamics within all time slots from frame to frame, we converted the temporal position *τ*_*k*_(*n*) of the *k*-th soliton in the *n*-th time slot into cylindrical coordinates following the relationship (*ρ*_k_(*n*), *ϕ*_k_(*n*)) = (*τ*_0_ + *τ*_k_(*n*), *n*2π/*N*), where *τ*_0_ is an arbitrary constant and *N* = 195 is the total number of time slots (see Figs. 2b and 4b). In each azimuthal “slice”, the amplitude of the pulsed signal is indicated by a colour map. When a soliton sequence consists of soliton molecules, the limited bandwidth of the PD would translate the soliton-pair (or triplet) structure into a single pulse with doubled (or tripled) amplitude compared with that of a single-soliton. See SI Section III for details.

### Diagnostic set-up and capacity

We used a 33-GHz-bandwidth PD for time-domain measurements, together with a 100 Gbit s^−1^ oscilloscope. This yielded a temporal resolution of ~15 ps, with a maximum recording time span of 2.5 ms that was limited by the oscilloscope memory. To reach longer recording times (5 ms), in a few cases (e.g., in Figs. 2b–d and 7b), we reduced the bandwidth to 16 GHz (50 Gbit s^−1^ sampling rate). For similar reasons, the recordings in Figs. 2b and 4b were at different frame rates (5 and 20 kHz), using the oscilloscope to take discrete shots of the round-trip signal during synthesis and dissociation.

### The DFT method

The DFT method provides a shot-by-shot mapping of the spectral profile in the time domain through the linear dispersive broadening of the multi-pulse structure over a sufficiently long optical fibre, following the time−frequency mapping relation^12^

$$\left| {A(z,t)} \right|^2 = \frac{2}{{\pi \beta _2z}}\left| {\widetilde{A}\left( {0,\frac{t}{{\beta _2z}}} \right)} \right|$$

where *A* is the optical field envelope, *Ã* is the Fourier-transformed spectrum, *β*_2_ is the group velocity dispersion and *z* is the propagation distance. For two-soliton structures, the soliton spacing *τ* can be directly retrieved from the interferometric fringe period Δ*t* in the DFT signal via $\tau = 2\pi \beta _2z/\Delta t$ while the phase relation can be inferred from the relative position of the fringe. In our experiment, the DFT signal is obtained by using a 3-km SMF-28 with a GVD of −22.5 ps^2^ km^−1^ (*D* = +17.65 ps km^−1^ nm^−1^) and detected with a 25-GHz PD, corresponding to spectral resolution δ*λ*_res_ = 1/(*B*|*D*|*z*) ≈ 0.7 nm at 1.55-µm wavelength, in which *B* is the PD bandwidth, *D* is the fibre dispersion and *z* is the fibre length. The optical bandwidth retrieved from the DFT signal is Δ*ν* ~ 12.5 THz or a Δ*λ* ~ 10 nm given by Δ*λ* = *T*/(|*D*|*z*) in which *T* is the span of the time slot. The maximum soliton spacing that can be retrieved by numerical fitting of the DFT signal is ~14 ps, which is limited by the PD bandwidth and the stretching ratio. The precision of the retrieved soliton spacing is affected by the signal-to-noise ratio of the PD and the intrinsic timing jitter of the oscilloscope. Given ~2-ps uncertainty in the fringe period of interferometric DFT signal, the precision of the retrieved soliton spacing should be <0.1 ps (for typical soliton spacing around ~10 ps).

### External addressing pulses

The addressing pulses launched into the laser cavity were generated by modulating a single-wavelength laser at 1550 nm using a programmable pulse-sequence generator (~200-ps pulse duration). The programmed pulse sequence followed a time grid that exactly matched the optomechanical lattice in the laser cavity, with 256 time slots and repeated at ~7.344 MHz (laser-cavity length different from that under global control), in order to precisely overlap with selected time slots. Ten evenly spaced slots within the programmed time grid were filled with addressing pulses, which were then amplified to ~20-W peak power in two amplifier stages. The launching of the addressing pulse was controlled by an optical switch (2-dB extinction ratio and 100-ns edge time). The input port was a 50/50 coupler, which was also used as an output coupler. The polarization state of the addressing pulses was adjusted so that they could be blocked by the inline polarizer in the cavity. In order to obtain a clean DFT signal without overlap from the addressing pulses, we inserted a 90/10 output coupler before the 50/50 output coupler.

## Supplementary information

SUPPLEMENTARY INFORMATION

Supplementary Movie 1: Soliton molecule synthesis

Supplementary Movie 2: Soliton molecule dissociation

## Data Availability

The data that support the plots within this paper and other findings of this study are available from the corresponding authors upon reasonable request.

## Code availability

The code used in this paper is available from the corresponding author upon reasonable request.
