# Supplementary material for: Synthesis and dissociation of soliton molecules in parallel optical-soliton reactors
Source: Light Sci Appl. 2021 Jun 7;10:120. doi: 10.1038/s41377-021-00558-x (PMC8184919; doi:10.1038/s41377-021-00558-x)
Supplement: Supplementary file 1 — SUPPLEMENTARY INFORMATION [file 41377_2021_558_MOESM1_ESM.pdf]

SUPPLEMENTARY INFORMATION for
"Synthesis and dissociation of soliton molecules in parallel optical-soliton reactors"

Wenbin He,[1,*,†] Meng Pang, [1,2,†] Dung-Han Yeh,[1,3] Jiapeng Huang,[1,3] and Philip St.J. Russell[1,3]

[1]*Max Planck Institute for the Science of Light, Staudtstrasse 2, 91058 Erlangen, Germany*

[2]*State Key Laboratory of High Field Laser Physics, Shanghai Institute of Optics and Fine Mechanics, Chinese Academy of Sciences, Shanghai, 201800, China (Present address)*

[3]*Department of Physics, Friedrich-Alexander-Universität, Staudtstrasse 2, 91058 Erlangen, Germany*

(Dated: 15 May 2021)

CONTENTS

I. Experiment setup details 2
II. Spectral analysis and DFT method 3
III. Cylindrical coordinate 8
IV. Reaction rate of soliton molecules 10
V. Stochastic soliton motion in reactions 12
VI. Numerical simulations 17
VII. Parallel reactions of soliton-triplet molecules 23
VIII. Mechanism of individual control 25
References 28

*Corresponding author. Email: wenbin.hit@hotmail.com
†These authors contributed equally to this work

I. EXPERIMENT SETUP DETAILS

The setup used in our experiment consists of an optoacoustically mode-locked laser[1–4] and external control setup that applies global- and individual-control, as sketched in Fig.S1. The mode-locked laser employs a unidirectional ring-fibre cavity with a gain section of erbium-doped fibre (EDF, 1.2-m length) pumped by two laser diodes at 980 nm (each with max. 900 mW power). A linear polariser and a few FPCs are used to initiate the mode-locking through nonlinear polarisation rotation (NPR)[5,6]. The optoacoustic mode-locking is enabled using a solid-core PCF described in system configuration. A tunable delay (TD) is used for adjusting the cavity length so as to allocate a specific harmonic of the cavity round-trip frequency into the acoustic resonance of the PCF. The total cavity length is ~ 20 m, which could be varied in different experiments, leading to different harmonic orders.

The global-control setup applies fast perturbations upon laser parameters, mainly the laser gain and cavity loss, so as to perturb the interactions between the multi-solitons in all time-slots while the harmonic mode-locking state (i.e. the optomechanical lattice) remains stable. The laser gain can be perturbed by changing the pump current of the pump-laser diodes using a function generator. The cavity loss can be perturbed by a fast variable optical attenuator (TA, Boston Applied Technologies, response time < 100 ns) inserted in the laser cavity. The relationship between the induced loss and the applied voltage is shown as inset of Fig.S1. The parallel reactions could also be initiated by tuning the delay-line or simply by rotating the FPCs. However, due to the mechanical nature of these methods, the whole process lasts too long to be clearly recorded, whereas the final stages of the reactions are similar to those under fast pump and cavity-loss perturbations.

The global-control technique needs to be implemented in cooperation with a properly chosen working point of the laser cavity. In addition, rise/fall time of the step-change in voltages applied to the laser diode and the attenuator are also carefully adjusted in order to trigger steady reactions without destabilizing the optomechanical lattice. For example, in order to realize soliton-molecule dissociation, the pump current needed to be reduced by $\sim 8\%$ with optimized fall edge of $50 - 100$ µs. If the pump current falls too fast, population oscillation would arise and eventually destabilizing the mode-locking state. Slower edge would cause, on the contrary, ultra-long dissociation time that may exceed the available time span of detection. Similar optimizations are also critical for cavity loss perturbations, which should be neither too fast nor too slow.

The individual-control technique is realized by externally launching a repetitive pulse pattern so as to precise address selected multi-soliton complexes and initiate the desired reactions. The external pulses generated by modulating a single-wavelength laser at 1550 nm using a programmable pulse-sequence generator ($\sim$ 200-ps pulse duration). The programmed pulse sequence followed a time grid that exactly matched the optomechanical lattice in the laser cavity, in order to precisely overlap with selected time-slots. The peak power of these external pulses in the cavity (after the 50/50 coupler) is attenuated to ~ 10 W, which is still

comparable to that of the intra-cavity soliton ($\sim$ 25 W), leading to non-trivial perturbations during their direct overlapping. The polarisation state of the addressing pulses was adjusted (using FPC-4 and -5) such that they could be blocked by the inline polariser (IP) in the cavity. In order to obtain a clean DFT signal without overlap from the addressing pulses, we inserted a 90/10 output coupler (not drawn) before the 50/50 output coupler in the laser cavity, which is unreachable for the addressing pulses launched into the unidirectional cavity.

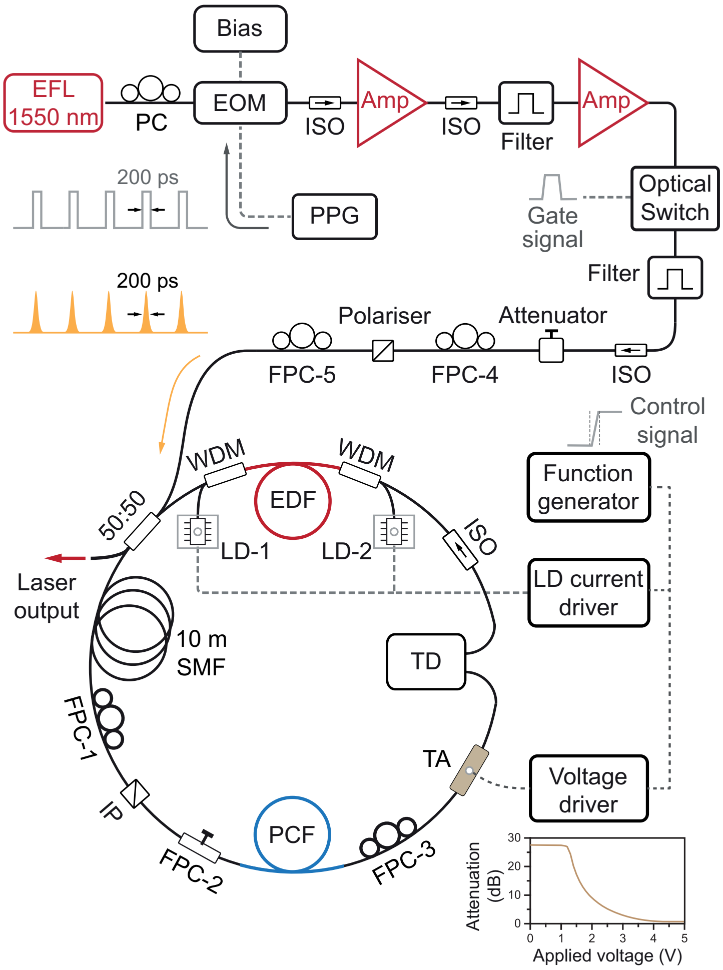

FIG. S1. Optoacoustically mode-locked soliton ring-fibre laser with external control setup. (See text for details)

II. SPECTRAL ANALYSIS AND DFT METHOD

The soliton molecule synthesis and dissociation can be simply interpreted as the transition between the long-range bound double solitons and the phase-locked soliton-pairs. A typical optical spectrum for long-range double solitons are shown in Fig.S2a. The 3-dB bandwidth is measured to be 2.9 nm, corresponding to $\sim$ 850 fs transform-limited pulse width, agreeing

with the autocorrelation trace (Fig.S2b). The spectral profile is mostly smooth with weak fringes appearing only in the vicinity of the dominant Kelly sideband (Fig.S2c), indicating that the two solitons have uncorrelated phase-relations while the binding is based on repulsive forces exerted by the dispersive waves[7]. The repulsion forces, which can be expressed in terms of carrier-frequency shift $\Delta\omega_{\mathrm{d}}$, depends on both the amplitude of the dominant sideband (A_{d}) and the relative phase between the sideband and the perturbed soliton ($\Delta\varphi_0$) as below[7]

$$\Delta\omega_{\mathrm{d}} \propto A_{\mathrm{d}} \exp(-h\Delta t)\Psi(\Delta\varphi_0) ,$$

in which h is a constant related to the bandwidth of the sideband (the decaying rate of the dispersive waves), Δt is the long-range spacing between the two solitons, and Ψ is a complicated oscillatory function that depends on the relative phase $\Delta\varphi_0$ as well as the dispersion and nonlinearity maps of the laser cavity. For synthesis, the global-control method that relies on pump power and cavity-loss perturbation mainly affects the repulsion force, either by reducing the dispersive wave amplitude A_{d} or dismantle the delicate phase-relation $\Delta\varphi_0$, such that acoustic-induced attraction force would lead to soliton collisions and eventually, the formation of soliton molecules.

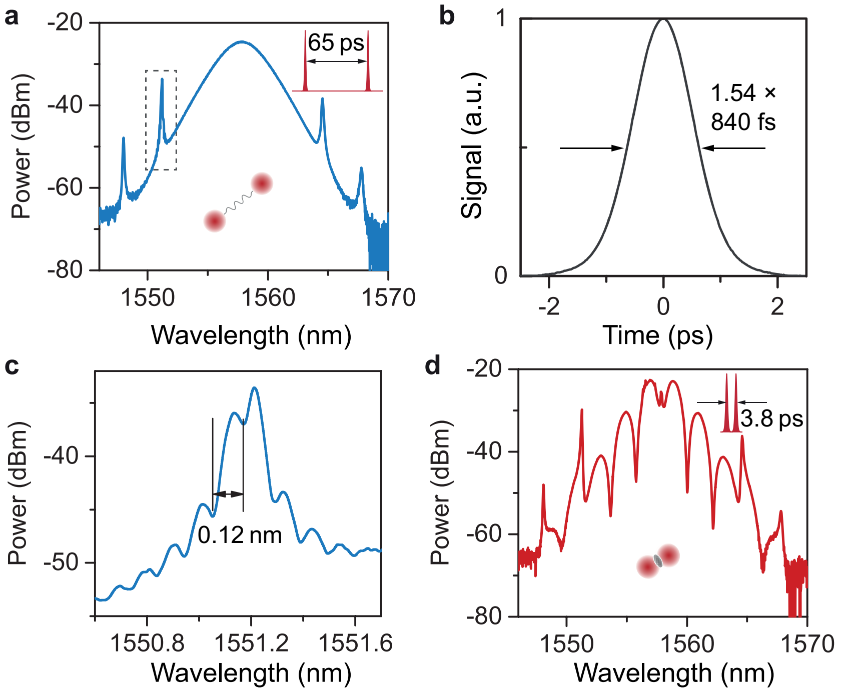

FIG. S2. **a** Optical spectrum of the mode-locked laser with all time-slots filled with long-range double-solitons. **b** Autocorrelation trace for individual solitons in the sequence. **c** Expanded view of the dominant Kelly sideband, featuring a weak fringe. **d** Optical spectrum of the mode-locked laser, after the synthesis, with all time-slots filled with soliton-pair molecules.

When the long-range double-solitons in all time-slots transit into soliton-pair molecules after synthesis, and the optical spectrum exhibit high-contrast interferometric fringes over

the entire profile (Fig.S2d). The narrow spike located at the central dip of the fringe indicates a weak CW components that appeared after the reaction, which can be eliminated by further adjusting of the working point (e.g. by rotating one of the FPC in the cavity.)

For simplicity we assume that the constituent two solitons have identical envelope $E(t)$[8], while spaced at τ and relative phase of φ_0. The total field can then be expressed as

$$E_{\text{tot}} = \Re\left\{[E(t) + E(t-\tau)e^{-i\varphi_0}]e^{-i\omega_0 t})\right\} ,$$

where $\Re$ stands for real-part of the complex form. Then the spectral interferogram of the complex envelope obtained from the Fourier transform should be

$$S(\omega-\omega_0) = |\tilde{E}(\omega-\omega_0)|^2\left[2 + 2\cos((\omega-\omega_0)\tau + \varphi_0)\right] ,$$

where ω_0 is the carrier frequency and $\tilde{E}(\omega-\omega_0)$ is Fourier transform of the soliton envelope. The inner spacing between the constituting solitons can then be determined from the optical spectrum to be ~ 3.8 ps and their relative phase to be $\sim \pi$.

A soliton-triplet molecule can be synthesized out of a soliton-pair molecule and a single-soliton, and reversely, dissociated into them. The optical spectrum for the long-range bound state of soliton-pair molecules and single-solitons also exhibit fringes due to the phase-locked soliton-pairs (see Fig.S3a), although with a lower contrast compared with that in Fig.S2d due to the coexistence of uncorrelated single-solitons. After the synthesis, the time-slots are occupied with soliton-triplet molecules, with the corresponding optical spectrum shown in Fig.S3b.

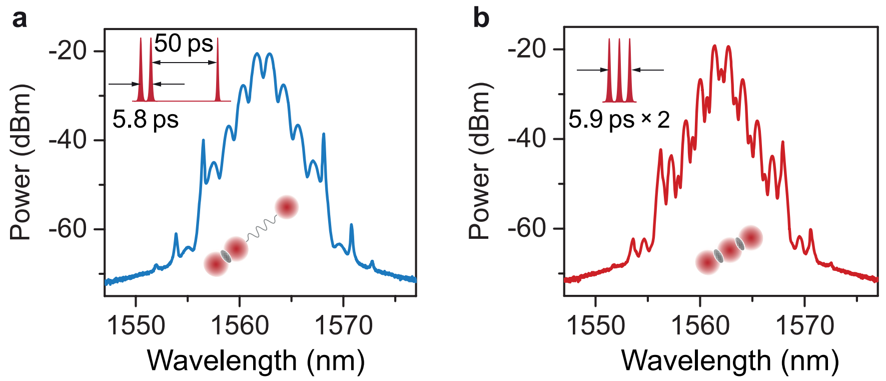

FIG. S3. **a** Optical spectrum of the mode-locked laser with all time-slots filled with the long-range bound state for soliton-pair molecules and single-solitons, as the preparation state of soliton-triplet molecule synthesis. **b** Optical spectrum of the mode-locked laser with all time-slots filled with soliton-triplet molecules, as the final state of the synthesis.

Here we still assume identical envelopes $E(t)$ for each individual solitons within the complexes, and the total field of a soliton-triplet can be described as:

$$E_{\text{tot}} = \Re\left\{[E(t+\tau_1)e^{i\varphi_1} + E(t) + E(t-\tau_2)e^{-i\varphi_2}]e^{-i\omega_0 t}\right\} ,$$

where τ_1 and τ_2, φ_1 and φ_2 are the inner spacing and relative phase between the consecutive solitons. Then the spectral interferogram of the complex envelope obtained from the Fourier transform should be

$$S(\omega-\omega_0) = |\tilde{E}(\omega-\omega_0)|^2 \{3 + 2\cos((\omega-\omega_0)\tau_1 + \varphi_1) \\ +2\cos((\omega-\omega_0)\tau_2 + \varphi_2) \\ +2\cos[(\omega-\omega_0)(\tau_1+\tau_2) + \varphi_1 + \varphi_2]\} \ ,$$

Accordingly, we can retrieve the inner spacing as $\tau_1 = \tau_2 = 5.9$ ps and their relative phase $\varphi_1 = \varphi_2 \approx \pi$ within the soliton-triplet molecule from the measured spectrum shown in Fig.S3b that exhibits multiple interleaved fringes.

To analyse the soliton trajectories during the dynamics processes, we need to retrieve the phase and spacing relation from the time-stretched DFT signal. For a typical DFT signal of a soliton-pair molecule, we can fit the time-domain interferometric signal with a sinusoidally modulated spectral profile, i.e.

$$S(t) = A\mathrm{sech}^2\left(\frac{t-t_0}{\Delta T}\right)\left[1 + \cos\left(\frac{2\pi}{\Delta t}(t-t_0) + \Delta\varphi\right)\right] \ .$$

Δt is the fringe period, which is related to the soliton spacing τ by the following relation

$$\Delta t = \frac{2\pi\beta_2 L}{\tau} \ ,$$

where β_2 and L are the dispersion and length of the SMF-28 fibre used for time-stretch DFT. $\Delta\varphi$ is readily the phase-relation between the solitons. A sech^2-shape is assumed for the spectral envelope of optical soliton, with amplitude of A and spectral width of $\Delta\omega \propto \Delta T/\beta_2 L$. A typical DFT signal of a bound soliton-pair and the corresponding fitting curve are given below in Fig.S4a, from which we can retrieve a soliton spacing of ~ 5.8 ps and phase-relation $\sim \pi$.

The fitting curve for a soliton triplet is complicated by compound interferences between three solitons, which can be given by

$$S(t) = A\mathrm{sech}^2\left(\frac{t-t_0}{\Delta T}\right)\left[\frac{3}{2} + \cos\left(\frac{2\pi}{\Delta t_1}(t-t_0) + \Delta\varphi_1\right) \\ + \cos\left(\frac{2\pi}{\Delta t_2}(t-t_0) + \Delta\varphi_2\right) \\ + \cos\left(\frac{2\pi}{\Delta t_1 + \Delta t_2}(t-t_0) + \Delta\varphi_1 + \Delta\varphi_2\right)\right] \ .$$

The DFT signal of a typical soliton triplet and the corresponding fitting curve are given in Fig.S4b. Note that this soliton triplet are selected from the same parallel reactors that hosts the soliton-pair molecule shown in Fig.S4a, therefore they share similar inter-soliton spacings (see below Fig.S18c that shows a few consecutive time-slots within the parallel reactions). We can readily notice that the DFT signal of the soliton triplet exhibits twice

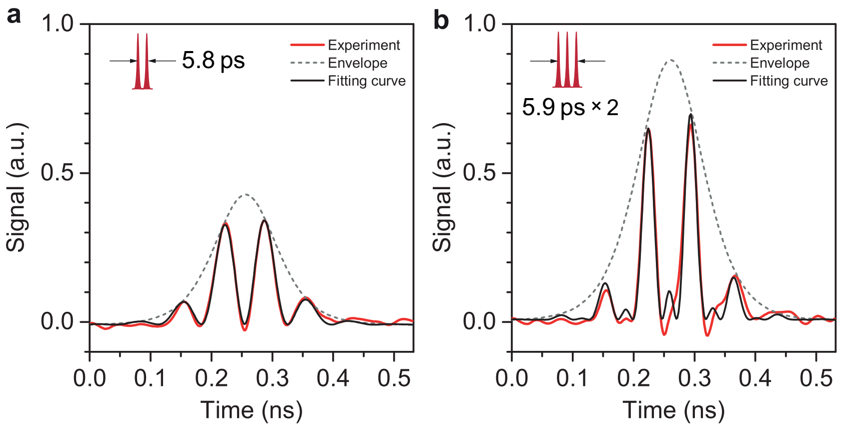

FIG. S4. DFT signal and retrieved interferogram out of curve fitting for analysing soliton molecule dynamics. **a** Typical DFT signal of a stable soliton-pair molecule. **b** Typical DFT signal of a stable soliton-triplet molecule. Solid red-curve: recorded signal, solid black curve: numerical fitting, grey dashed line: spectral envelope.

the envelope amplitude while having sharper fringes and weak bumps in between, which can hardly be recorded genuinely by the current photodetector. In general, the curve fitting for soliton-triplet DFT signal is more challenging than that for soliton-pair, due to not only the increased numbers of fitting parameters, but also the symmetric form of the fitting curve that can give non-unique fitting parameters (flipping the value of Δt_1 and Δt_2, $\Delta\varphi_1$ and $\Delta\varphi_2$ would result in the same interferogram). Particularly, in case of radical collision and repulsions (see Section V), we can no longer track the exact order the interacting solitons from the DFT signal while the consecutive spacing and phase-relation become uncertain. Currently, we can only retrieve simple and smoothly changing three-soliton dynamic from the DFT signal in which a bound soliton-pair already exist within the structure and was only slightly perturbed during the dynamic processes. We illustrate below as example in Fig.S5 the comparisons between the original DFT signal and the retrieved interferogram through curve fitting below for soliton-triplet dynamics (trajectories given in Fig.3f and Fig.6b), which exhibit good agreements (except for top region of Fig.S5a and bottom region of Fig.S5b where the dense fringes exhibit weak contrast.)

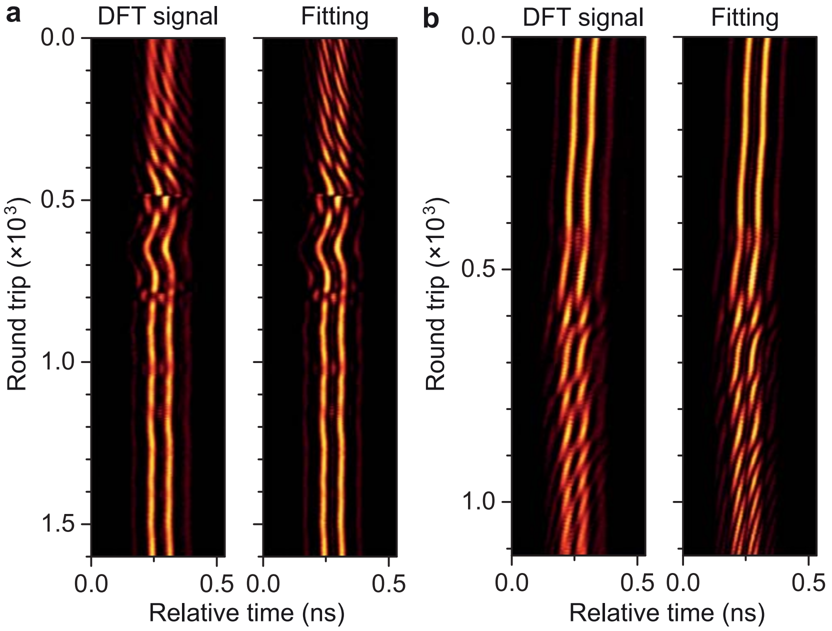

FIG. S5. Original DFT signal and retrieved interferogram out of curve fitting for analysing soliton-triplets dynamics. **a** Soliton-triplet molecule synthesis, with the retrieved trajectory given in Fig.3f. **b** Soliton-triplet molecule dissociation, with the retrieved trajectory given in Fig.6b.

III. CYLINDRICAL COORDINATE

In a cylindrical coordinate (ρ, ϕ, z), we converted the temporal position $\tau_k(n)$ of the kth soliton in the nth time-slot following the relationship $(\rho_k(n), \phi_k(n)) = (\tau_0 + \tau_k(n),\ 2\pi n/N)$, where τ_0 is an arbitrary constant and N is the total number of time-slots. In each azimuthal "slice", the amplitudes of each soliton belongs to the z-axis and are indicated by a colour-map. In this way the relative positions of solitonic elements in all time-slots of the ring-cavity are then clearly presented. As a simple demonstration, we plot the preparation state for soliton-triplet molecule synthesis in the cylindrical coordinate as shown in Fig.S6, with a single-soliton and a soliton-pair molecule in each of the 160 time-slots (except a few time-slots that hosts only soliton-pair molecules). Each division occupies an azimuthal angle of $\delta\phi_{\mathrm{div}} = 2\pi/N \approx 0.04$ rad (highly exaggerated by the white-line highlighting), while the recorded signal within each time-slot are plotted along the radial axis. The pulsed signal with twice amplitude indicates a soliton molecule, as a result of the limited PD bandwidth. The white circle marked the span of one mechanical cycle ($\Delta\rho_{\mathrm{slot}} \sim 0.532$ ns in time or ~ 10 cm in space), which is also the length of each time-slot.

We employ cylindrical coordinate to illustrate the full recordings (with periodic snapshots) of soliton molecule synthesis (Fig.2b and Movie 1) and dissociation (Fig.4b and Movie 2). All frames are precisely aligned so that a time-slot that accommodates specific reacting solitons remained in the same azimuthal angle $\phi(n)$ from frame to frame. In addition, the

DFT signal of specific time-slot are aligned to same azimuthal angle $\phi(n)$ as that in the time-domain signal.

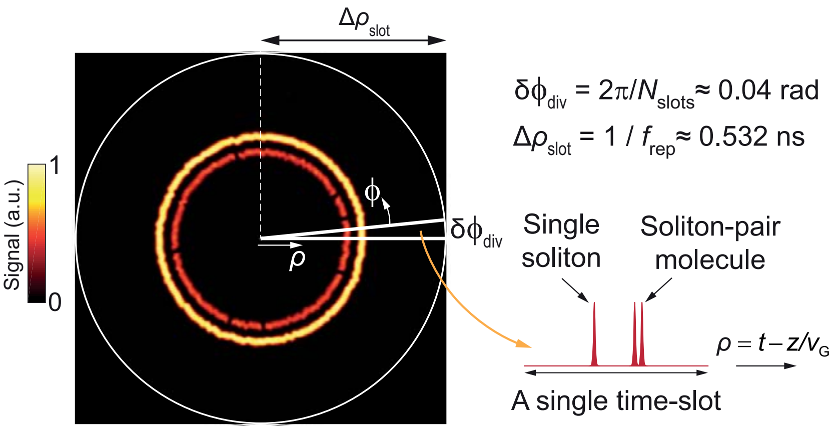

FIG. S6. A soliton supramolecule plotted in cylindrical coordinate, which consists of 160 divisions that accommodate both single solitons and soliton-pair molecules.

In Movie 1 (as well as in Fig.2b in the main text), we can see that only one time-slot is prepared with a single soliton as reference, which appeared as a lower peak (colour-map defined in Fig.S6) and remained unchanged till the completion of the synthesis. We highlight the reference slot with the single soliton in a zoom-in figure below in Fig.S7. As marked by dashed circle, the reference slot only hosts one soliton before the reaction which persist till the end of the reaction with a slightly different balanced position.

In Movie 2, all time-slots are prepared with soliton-pair molecules. Some of the molecules however end up with losing one of the constituting soliton after dissociation, due to radical repulsions similar to that illustrated in Panel (iv) of Fig.5a (See details below in Section V). These time-slots, with only half the energy of two-soliton slots, appeared as a few dimmed slices in the DFT signal in the final state.

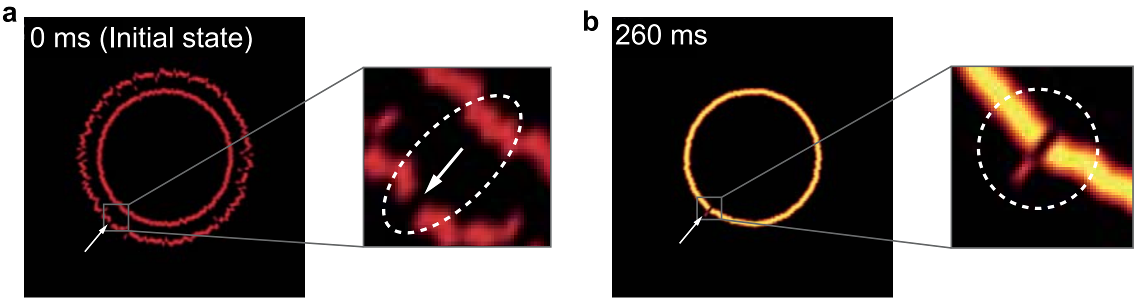

FIG. S7. **a** The initial state for two-soliton interaction, while one time slot only consist one soliton as marked in dashed circle in the zoom-in figure. **b** After the reaction completed, the one-soliton slot remains (marked in dashed circle) while other time-slots host soliton molecules.

IV. REACTION RATE OF SOLITON MOLECULES

Resembling chemical reactions, the formation of soliton molecules requires multiple collisions of individual solitons in the trapping potentials before an effective collision occurred that leads to establishment of the molecular bond. Using the frame-by-frame recording of the long-term synthesis (one example shown in Supplementary Movie 1), we can roughly estimate the synthesis rate of the soliton molecule as well as collision rate of interacting soliton during the reaction. As shown in panel (iii) of Fig.3a, the separated pulses in each time-slot merge into a single pulse with doubled amplitude (due to the limited PD bandwidth,) and then repel each other again into two pulses. We regard one such action as one "collision". After many times of such collisions, they eventually merge into a stable soliton molecule (a single-peak signal) with stable fringe in the DFT single. We repeat the synthesis experiment for several times, and plot the collision number as well as the soliton molecule number versus round-trip number (or time) during the synthesis. Two examples are shown in Fig.S8. We revealed that although the collision rate and synthesis rate seems to vary from experiment to experiment (probably sensitive to discrepancies in the initial states and perturbation strength from time to time), their correlation exhibits a quasi-linear relationship, reminiscent of classical chemical kinetics in which the rate of reaction is proportional to the collision frequency of the reactants. Although the soliton reactions that occurred between pre-sorted and paired solitons within the parallel reactors do not fully mirror the real chemical reactions in which all reactants freely collide with each other, the experiment result still provide interesting insight into the statistical properties of soliton interactions and can be further improved and modified to host various reaction conditions.

The parallel dissociation of soliton molecules under global control is found to follow an exponentially-decaying profile, which can be described by the first-order reaction model with a rate equation as

$$\frac{dN}{dt} = -kN \ ,$$

where N is the instantaneous molecule number during the reaction at time t. The general solution is an exponential decay with a "half-life" of $\ln(2)/k$.

In our analysis, a soliton molecule is regarded as completely dissociated when the two solitons are repelled beyond a significantly large spacing (i.e. 14 ps in the article) than the molecular spacing. The criterion is rather arbitrary within a reasonable range. We plot the results under different spacing criteria in Fig.S9. We can note that, since the initial repelling between the solitons at short-range is generally fast, the exact decaying curve varied only slightly under different criterion as shown in Fig.S9a. The spacing evolution becomes rather slow later at long range due to weakened interaction. Consequently, the formation rate of long-range double-solitons varied significantly under different criteria, as shown in Fig.S9b.

Incidentally, since soliton molecule dissociation is generally much faster then their synthesis, detailed studies on soliton motions using DFT signal in this work are mostly based

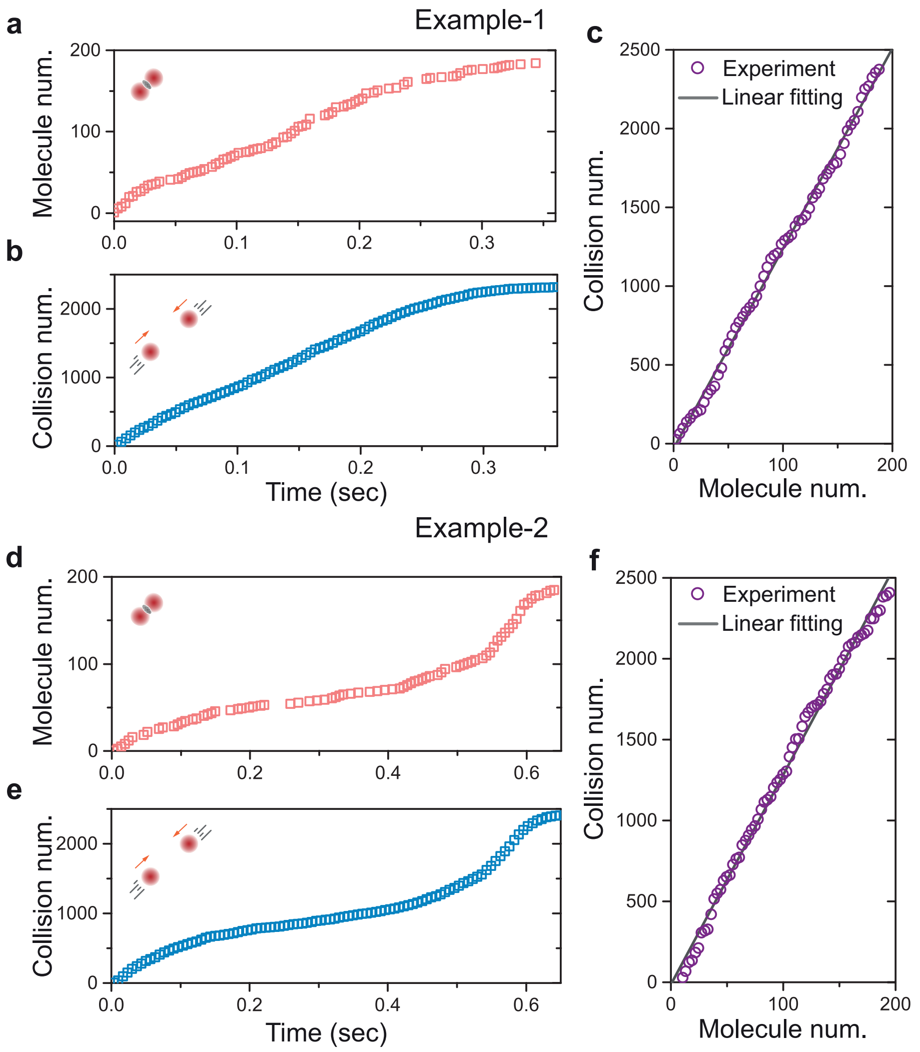

FIG. S8. Correlation between synthesis rate and collision rate of soliton molecules. Results from two synthesis experiments under the same global-control parameter ($\sim 8\%$ pump power change) are shown as Example-1 and -2. **a**, **d** Soliton molecule number over all parallel reactors that gradually accumulated over time during the synthesis. **b**, **e** Total soliton collision number accumulated over all parallel reactors. **c**, **f** Cross-plots of collision number versus the soliton molecule number during each synthesis, both exhibiting quasi-linear proportionality.

on results during dissociation in which the entire process over all time slots can be fully recorded round-trip by round-trip. Meanwhile, effective collisions between solitons during the parallel synthesis can distribute over hundreds of milliseconds (see Fig.2b), and only a small fraction of them can be recorded with high resolution (see Fig.2d). Nevertheless, using limited results obtained from synthesis experiment, we can still realized that soliton motions in both processes share similar features, including random-walk-like motion and repulsions at intimate spacings.

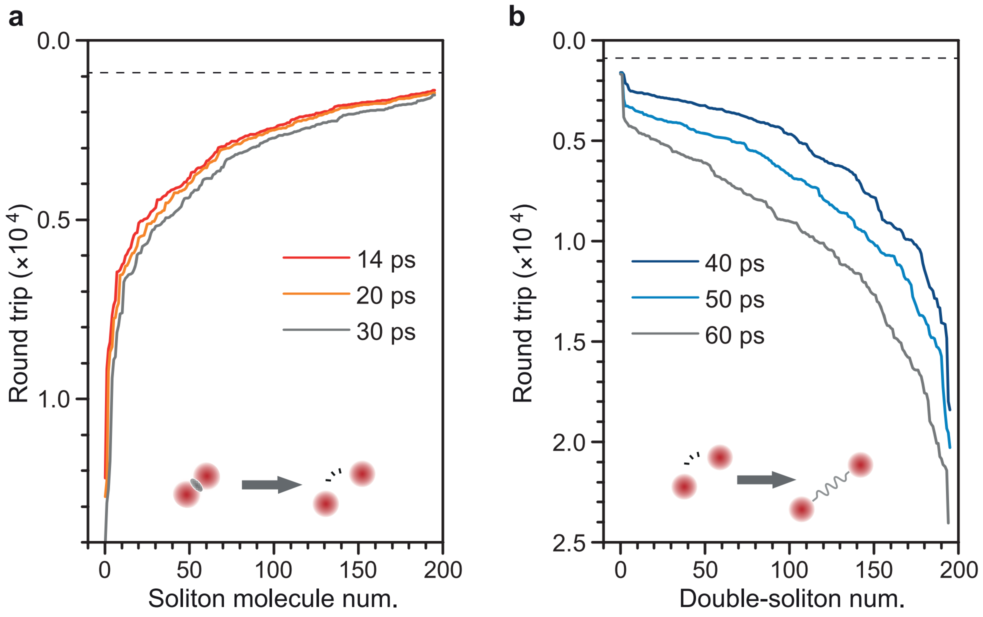

FIG. S9. **a** Soliton-molecule number decaying and **b** long-range double-soliton number increasing over consecutive round trips under different spacing criteria. Different curve colours are used to mark counting results under different spacing criteria. The dashed lines in both plots indicate the time at which the perturbation occurred.

V. STOCHASTIC SOLITON MOTION IN REACTIONS

Random-walk-like soliton trajectories. The motion of the solitons during synthesis and dissociation are found to be highly stochastic, resembling one-dimensional random walk with fixed length of motion step. The random-walk-like motion is probably a result of phase-dependent interactions between the solitons due to the oscillatory wing-structures as well as randomly-excited dispersive waves. The soliton interactions that depends on their relative phase leads to changes in spacing which in turn leads to changes in relative phases, causing random and oscillatory motions. In addition, non-solitonic wave perturbations, including dispersive waves and weak CW background may also contribute to the stochasticity of the soliton motions.

Here we provide a few examples (see Fig.S10a – c) observed during soliton molecule dissociation, where the step-wise evolutions of soliton spacing are prominent. As shown in Fig.S10c, a minimum step (spacing change) is $\sim$ 0.6 ps over $\sim$ 50 round trips. To highlight the similarity, we numerically generated a one-dimensional random walk trajectory in Fig.S10d, with step length of 1 and a random direction ($+1$ or -1). The evolution steps are found to be shared over all parallel reactors during the parallel reactions, while the step length varied slightly under different cavity parameters. We suspect that the step length is related to the location of the dominant Kelly sideband relative to the spectral centre, i.e. the beat note between the dominant dispersive wave frequency and the soliton carrier

frequency. Similar random-walk-like motion is also reproduced in our simulation results (see Section VI).

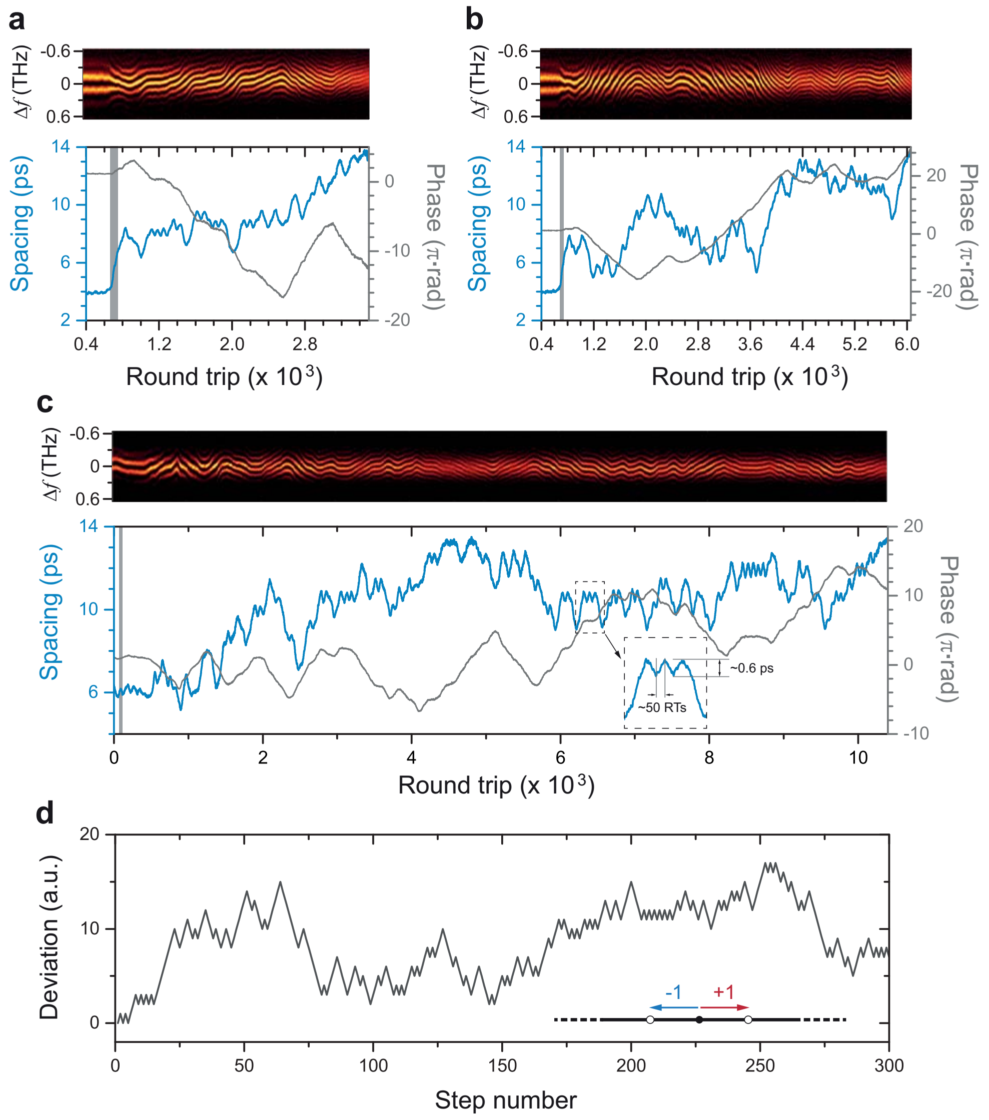

FIG. S10. Random-walk-like motion during soliton-pair molecule dissociation. **a** – **c** DFT signal of dissociation of soliton-pair molecules in three time-slots. The grey vertical bar indicate the time of cavity perturbation. The initial soliton spacing is 4 ps in **a**, **b** and 6 ps in **c**. **d** A numerically generated 1D random walk over 300 steps, with fixed step length of 1 and random direction (+1 or −1).

Despite their similarity, a few prominent differences exist between the soliton motions and generic random-walk paths. Firstly, two boundaries exist for soliton spacing during the reactions. The lower boundary is the molecular spacing, below which the soliton interaction become universally repulsive. The higher one is the long-range binding spacing, toward which the soliton spacing no longer follow a step-wise evolution. In addition, a few

metastable spacings could exist, at which the interacting solitons can temporally reside.

Soliton repulsions at intimate spacing. The interacting solitons during reactions, despite resembling random-walk trajectories, would universally run into radical repulsion whenever their spacing reached below the original molecular-spacing. Such repulsion could quickly push them apart and even cause immediate the dissociation. The strength of repulsion would vary from time-slot to time-slot in the parallel reactors and multiple repulsions could occur within a single trajectory. In addition to the two examples given in the article (Fig.5e and 5f), soliton repulsion could be quite mild, as the example shown in Fig.S11a, which only lead to slow diverging of the two solitons; or rather radical, as the example shown in Fig.S11b, which lead to extinction of both solitons after the sharp diverging.

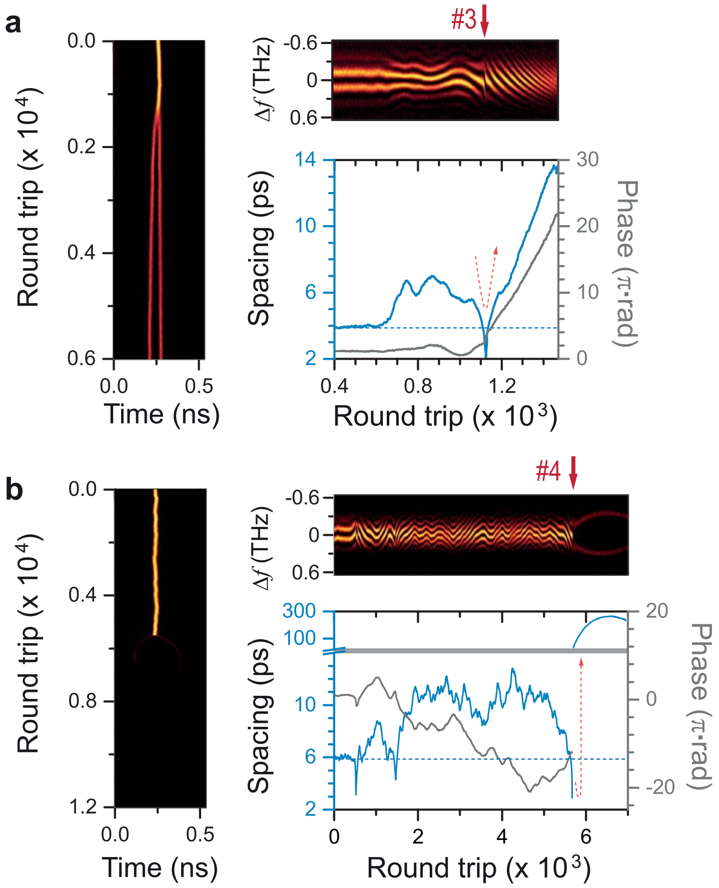

FIG. S11. Two examples of soliton-pair molecule dissociation terminated by soliton repulsion. **a** The time-domain trace (left), the DFT signal (right-top) and the retrieved soliton trajectory (right-bottom) of a dissociation that involve a mild repulsion marked by the red arrow. **b** Dissociation terminated by a repulsion that lead to fast diverging and then extinction of the interacting solitons.

The expanded view at the DFT signal during the soliton repulsions listed above and in article (marked by red arrows #1 – #4 in those figures) are illustrated below (Fig.S12a) in parallel in the order of repulsion strength. We can reveal that in all these events, the two

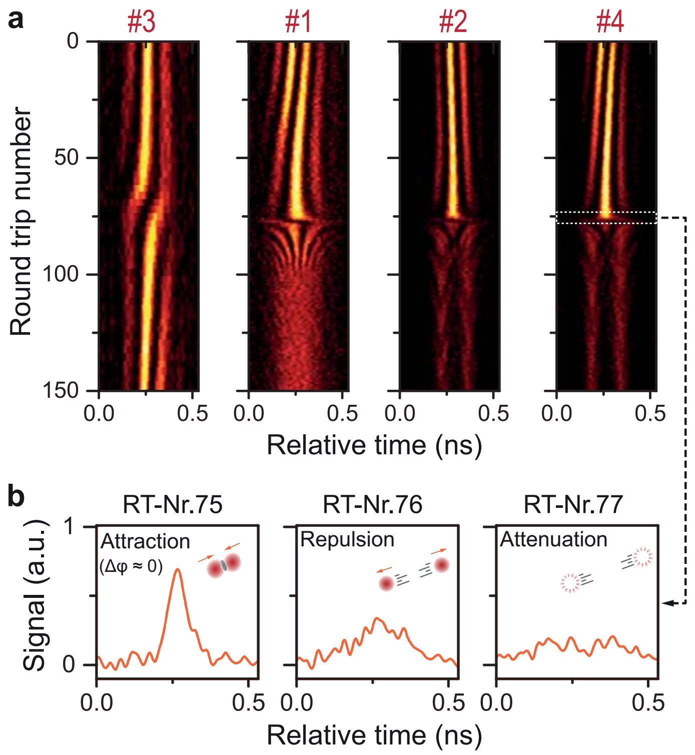

FIG. S12. **a** Expanded views of the DFT signal at regions where soliton repulsions occurred during the dissociation marked as #1 – #4 in Fig.6 and S11. In each case, we plot the DFT signal over 150 consecutive round trips where the repulsions occurred in the middle of selected time-span. **b** DFT signal over 3 consecutive round trips at which radical repulsion occurred in case #4.

solitons first drifted toward an in-phase relation (with a peak in centre of the DFT interferogram) as their spacing sharply dropped, in accordance to the conventional theory that in-phase relation induces attraction. Then, such attraction force immediately transformed into radical repulsion that prevent further merging of two solitons. The repulsion of two optical solitons in optical fibres at intimate spacing has been noticed since decades[9], while the exact mechanism behind this phenomenon remains unclear. It seems that a mutual shift in the carrier-frequencies of the soliton-pair has occurred that quickly shifted the phase relation from in-phase to out-of-phase, turning the soliton interaction into strong repulsion. The relative-phase shift is given by $\Delta\varphi = 2\pi\Delta\nu\Delta t$, where $\Delta\nu$ is the carrier-frequency shift of the soliton-pair and Δt is their spacing. We can estimate that the frequency shift in our case should be in the order of 0.1 THz so as to have a π-phase shift.

Soliton self-frequency shift due to Raman effects[10] has been suspected to cause such shift of phase-relation. However, we found in numerical analysis that strong repulsion can occur regardless of our choice of Raman effect being considered or not (see a similar radical repulsions reproduced in our simulation results shown in Fig.S17c.). Therefore, we suspect that dissipative factors such as gain saturation, gain filtering effect, and peak-power clamping

effect provided by the NPR-effect[3,11] should be accounted for such soliton repulsion that prevented an enhanced soliton amplitude.

Occasionally, the soliton repulsion could occur so radically that the DFT interferogram would change significantly within even one round-trip. We plot the detailed DFT signal in case #4 signal during the repulsion in Fig.S12b, where we can reveal that within only 3 round-trips, the two solitons turn from intimate attraction into nearly total extinction. The exact soliton motion is therefore out of the capability of the current DFT method.

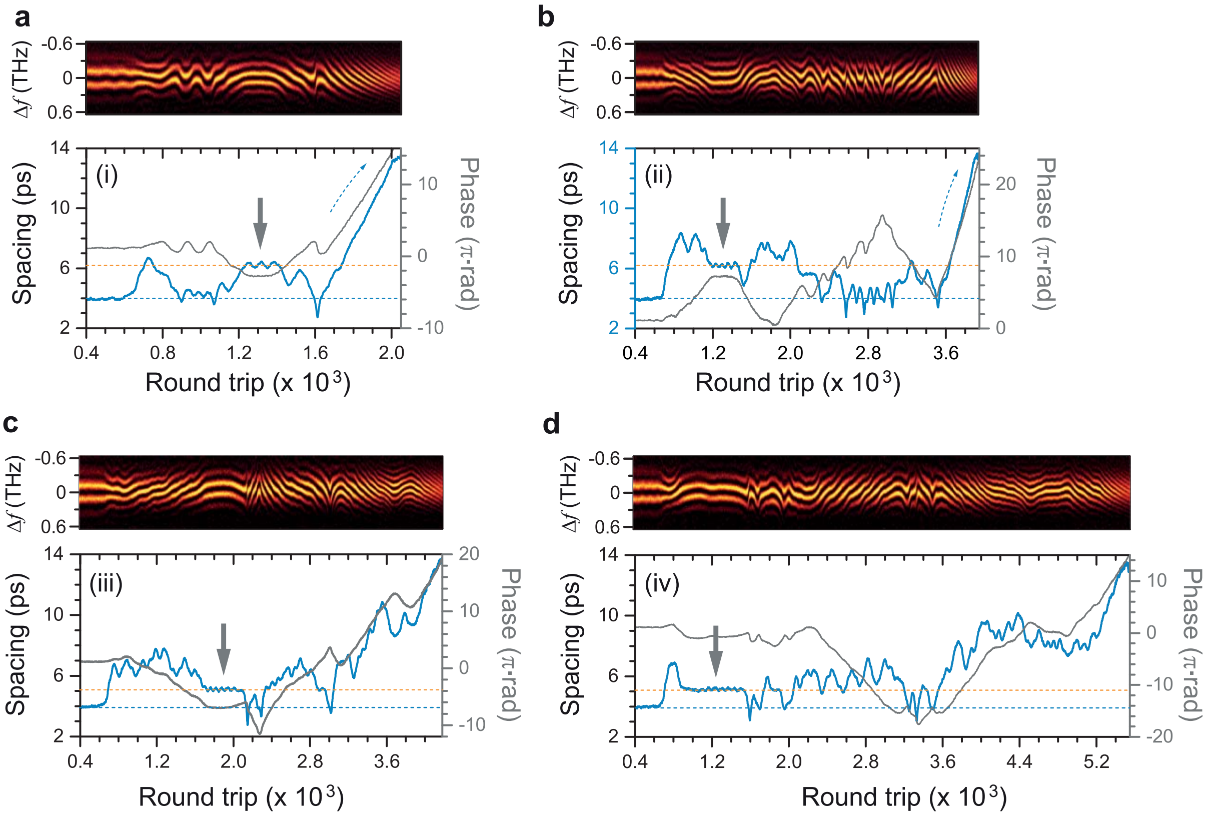

FIG. S13. DFT signal and retrieved spacing/phase-relations during dissociation in four parallel time-slots which exhibit metastable states (marked by grey arrows). **a** and **b** shared the same metastable spacing at 6.2 ps, while **c** and **d** shared the same metastable spacing at 5 ps. The initial molecular spacing is indicated by the blue dashed line, while the metastable spacings are indicated by the yellow dashed lines.

Metastable states of interacting solitons. The random-walk-like trajectories during the soliton reactions occasionally consisting of an additional feature that the interacting solitons would temporally reside at some metastable state, with fixed or only weakly oscillatory spacing and phase, as we spotted within the massive events occurred in the parallel reactors. Moreover, the same metastable states could appear in different reactors during one parallel-reaction process, which suggests that they might correspond to some weak attractors of the current nonlinear system. As an example, we provide here in Fig.S13 a few recorded

dissociation trajectories that exhibit metastable states. The metastable regions within the dissociation trajectories in each time-slot are marked by grey arrows. We can notice that the trajectories in Fig.S13a and b share the same metastable spacing at $\sim$ 6.2 ps, while Fig.S13c and d share the metastable state at $\sim$ 5 ps (the metastable spacings are marked by the yellow dashed lines).

VI. NUMERICAL SIMULATIONS

Simulation settings. In order to further understand the dynamics of soliton molecules during their synthesis and dissociation, we build up a Python-based numerical code to simulate the multi-soliton propagation in a mode-locked laser cavity. The propagation in fibre sections are based on the generalized scalar NLSE as below:

$$\frac{\partial A}{\partial z} = -i\frac{\beta_2}{2}\frac{\partial^2 A}{\partial t^2} + \frac{\beta_3}{6}\frac{\partial^3 A}{\partial t^3} + i\gamma|A|^2 A + \frac{g}{2}A + \frac{g}{2\Omega_{\mathrm{g}}^2}\frac{\partial^2 A}{\partial t^2} \ ,$$

in which z is the propagation distance in each fibre section, t is the relative time in the moving frame, $A(z,t)$ is the field envelope of the multi-solitons such that the absolute square of A is readily the power profile. We consider up to third-order dispersion of each fibre section, which correspond to the first two terms on the RHS. The third term denotes the SPM effect, with γ being the nonlinear parameter. For SMF-28, $\beta_2 = -22.5$ ps^2 km^{-1}, $\beta_3 = 0.12$ ps^3 km^{-1}, and $\gamma = 1.3$ km^{-1} W^{-1}, while for PCF, $\beta_2 = -157$ ps^2 km^{-1}, $\beta_3 \approx 0.1$ ps^3 km^{-1}, and $\gamma = 33$ km^{-1} W^{-1}. In the simulation we consider β_3 only for these two sections due to their dominant length in the cavity. For EDF $\beta_2 = +77$ ps^2 km^{-1} and $\gamma = 9.3\ km^{-1}\ W^{-1}$, and for DCF $\beta_2 = +121$ ps^2 km^{-1}, $\gamma = 3.4$ km^{-1} W^{-1}. For the EDF section, we consider a saturable gain parameter with limited gain bandwidth, which is denoted by the last two terms on RHS. The saturable gain coefficient g can be expressed as

$$g = g_0 \exp(-\frac{\int |A|^2 dt}{E_{\mathrm{sat}}}) \ ,$$

where g_0 is the small-signal gain coefficient and E_{sat} is the saturation energy. In the parameter setting, the total gain is set to be slightly larger than the total cavity loss while the steady-state gain will be saturated and exactly balanced out the cavity loss. The gain filtering is approximated with a parabolic profile with a 20-nm bandwidth centred at 1560 nm. The saturable absorption is based on NPR in experiments while in simulation we use a simplified power-dependent transmission function

$$f(t) = 1 - l_0 \exp(-\frac{|A(t)|^2}{P_{\mathrm{sat}}}) \ ,$$

where l_0 is the low-intensity loss (modulation depth) and is set to be 0.1 dB, while P_{sat} is saturation power and is set to be 10 W.

The length of each fibre section follows the experiment set-up used in global control (for individual-control experiment, an additional SMF-28 sections will be needed.) The total cavity length is ~ 20 m (mostly SMF-28), while the average dispersion is ~ -22.24 ps^2 km^{-1}. We make use of the symmetrized split-step Fourier transform method to solve the NLSE for the propagating pulses in the fibre sections, while other components are regarded as lumped element in each loop of propagation. The time window used in our simulation is 512 ps, which is digitized with 2^{13} data points, while a finer resolution only has trivial effects upon the results. We performed simulations on both soliton-molecule synthesis and dissociation and the results highly agreed our experiment observations with many reproduced features. We demonstrate some of the simulation results below to highlight the key features of the soliton dynamics.

Soliton molecule synthesis: simulation results. In order to simulate the formation a soliton molecule, we first set two pulses that are initially spaced at 60 ps as the input. Then the two pulses evolve separately in the cavity to gain a soliton profile. Then we introduce an effective trapping potential by inserting a refractive index modulation as an lumped element in the cavity. This trapping potential provides the re-timing force originally induced by the acoustic waves in the PCF. Due to the presence of this trapping potential, the two solitons would slide toward each other and start to interact following a stochastic trajectory. The modulation depth of the refractive index potential will directly affect the collision rate of the solitons and eventually, the total reaction time of the solitons. We set the modulation depth in the range of 10^{-7} to 10^{-8}, which is in the same order of magnitude of the acoustically induced index change in our experiments[3]. The reaction time of the solitons in the simulation result generally agreed with our experiments, both in the order ms time-length (or $\sim 10^4$ round-trips).

A typical dynamics of soliton-molecule formation is shown below in Fig.S14. We can readily see from the time-domain soliton motion (Fig.S14a) that multiple times of collisions (marked by white arrows) occurred before a bound state with fixed spacing and π-phase relation is established. The corresponding spectral-domain evolution (Fig.S14b) features a varying spectral fringe, which is unfolded by the DFT signal in the experiments. The soliton molecule in the end of the interaction has stable spacing of ~ 3 ps and phase-difference of π, as we can seen from the temporal profile in Fig.S14c and the optical spectrum in d.

An important feature of the soliton molecule out of the synthesis is that it has a different group velocity from individual solitons. Thus its balanced position in the trapping potential would be slightly off the centre, which is in accordance to our experimental observations as shown in Fig.S7b. We noticed that this feature is very likely to be caused by higher-order dispersions in optical fibres that caused asymmetric Kelly sidebands generation, leading to a slight frequency shift of the resultant soliton molecule. Other factors that leads to spectral asymmetry (e.g. the asymmetric gain profile) may also contribute to this effect in real experiments. Similar phenomenon has also been noticed in a recent work based on

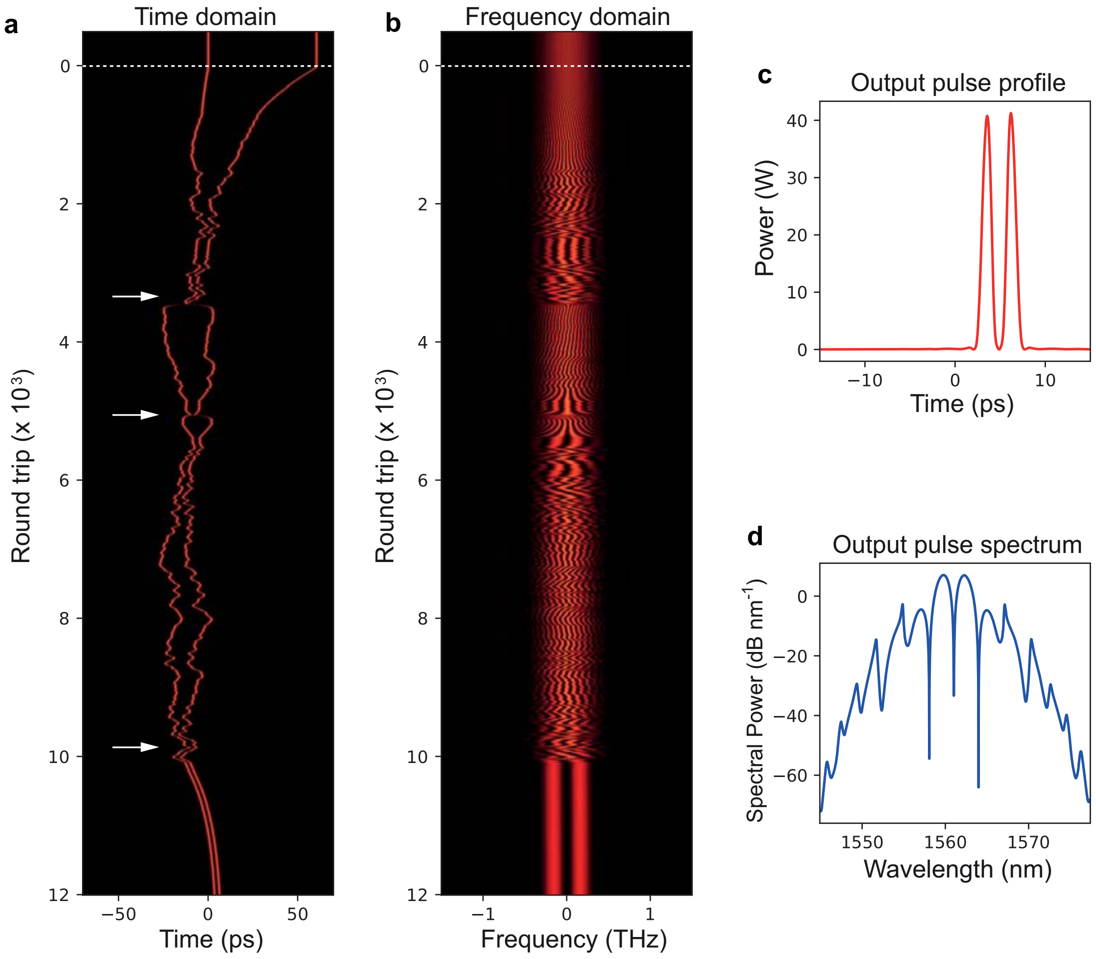

FIG. S14. Numerical simulation of soliton molecule synthesis. The initial state includes two solitons separated at a 60-ps spacing, then an effective trapping potential is added to the cavity (indicated by the white dashed line) to induce soliton collisions. **a**, **b** Soliton dynamics in time and frequency domain. The soliton collisions are marked by the white arrows, the last one being effective. The dashed horizontal line marks the insertion of the index potential. **c** The temporal profile of the stable soliton molecule out of the simulated synthesis. **d** The simulated spectrum of stable soliton molecule.

microresonators (see Ref.[12]). We expect that this topic can be further investigated with an improved numerical model.

Many detailed features that were observed in the experiments (see Section V) have been reproduced in the simulations. Using the soliton trajectory shown in Fig.S14a, we plot in Fig.S15a the soliton spacing and phase difference over the entire synthesis. This result can be directly compared with the experimental results in shown in Fig.3 of the main text. Three parts of these trajectory is zoomed-in in Fig.S15b – d. In Fig.S15b, random-walk-like motion of interacting solitons at intermediate spacings ($\sim$ 10 ps) can be noticed. In

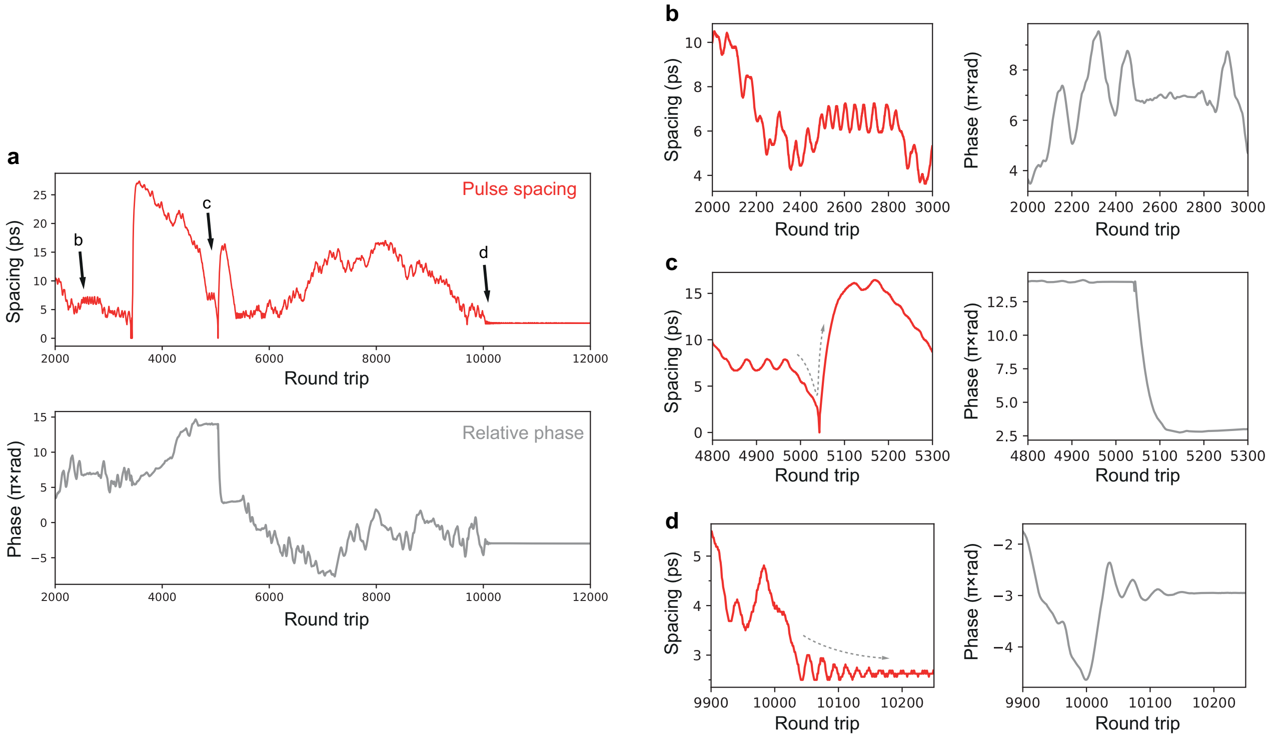

FIG. S15. **a** Detailed trajectories of solitons during molecule synthesis dynamics, including the soliton spacing (red curve) and the relative phase (grey curve). Note that here the soliton spacing is directly obtained using the temporal profile in Fig.S14a. Three features are highlighted by the arrows and the corresponding zoom-in plots are shown in **b** – **d**. **b** The random-walk-like trajectory of the soliton spacing. **c** A radical repulsion between the solitons occurred at a spacing below the molecular spacing. **d** A damping oscillation of soliton spacing shortly before the formation of stable soliton molecule.

Fig.S15c, a radical repulsion occurred at the molecular spacing can be noticed. This radical repulsions are likely to be caused by dissipative factors that prevent excessive peak power out of merged solitons. Incidentally, we can safely exclude the factor of Raman-shift in such radical repulsions as it does not play a significant role in this model. Finally, in Fig.S15d, we can notice a damping oscillation of the soliton spacing and relative phase before they settle down to a stable bound-state, which agreed with our observations shown in Fig.3b.

The parallel reactions in experiments shows highly stochastic processes that differs from time-slot to time-slot. This diversity of trajectories can be dominantly attributed to the different initial conditions of the interacting solitons. To demonstrate this effect, we perform two simulations with identical parameters except of a slight difference in the initial phase relation (by 0.1 rad). The results out of these two simulations are shown in Fig.S16. We can see that these two processes are dramatically different. The first synthesis shown in Fig.S16a is relatively fast and involves only one collision, while the second example shown in Fig.S16c takes nearly 10 collisions before entering the stable state. In real experiments,

all the solitons are uncorrelated in phase before the interactions, which would naturally lead to highly diverse trajectory even under the same cavity parameters, let alone the fact that the inevitable cavity noises will introduce additional randomness to the dynamics.

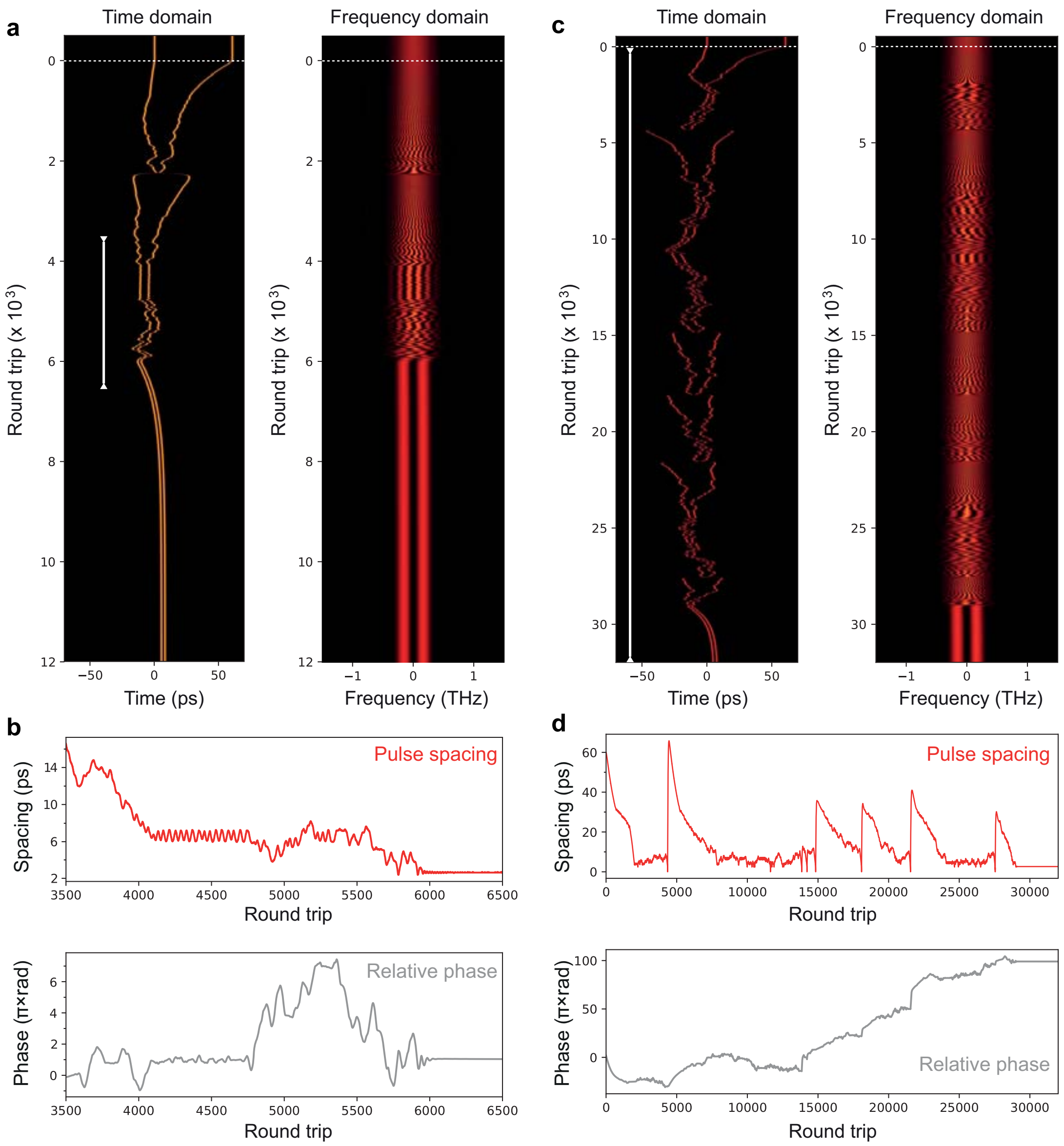

FIG. S16. Simulation results of two synthesis dynamics with identical parameters except for a slight (0.1 rad) difference in the initial phase relation. **a** The time (left) and spectral (right) domain evolution of the interacting solitons of the first examples. The white dashed line indicates the starting of the reaction. **b** The corresponding trajectories of the interacting solitons in terms of their spacing and relative phase. The vertical line in **a** marks the region for plotting the spacing and relative phase. The second example shown in **c** and **d** follows the same captions as **a** and **b**.

Soliton molecule dissociation: simulation results. Similar to our experimental observations, we notice in simulation that the break-up of a soliton molecule is much easier compared with the formation process. Given a stable soliton molecule as a initial state, a slight change of cavity parameter can lead to immediate break-up of the molecule. We show in Fig.S17 three simulation results of dissociation, with time- and frequency-domain evolution given in Fig.S17a, c, and e, while the corresponding spacing and relative phase evolution are given in Fig.S17b, d, and f. The simulation parameters are identical in these simulations except for a trivial difference (10^{-3}) in the change of the saturation energy. The trajectories in these three examples are highly stochastic, while the features present in synthesis also appeared in dissociation. In Fig.S17a, the two solitons gradually dissociate following a random-walk-like motion. In Fig.S17c, a radical repulsion between the two solitons immediately break-up the soliton molecule (marked by arrows in the bottom-left panel). The inset that gives an expanded view over the repulsion region (marked by the dashed boxes) can be compared with the DFT signal shown in Fig.S12. The strength of the repulsions can vary significantly in different collisions. In Fig.S17e, we can notice a soliton repulsion that is stronger than the case in Fig.S17b. Moreover, we can observed a metastable state at which the two solitons maintained for about 1000 round trips (with an expanded view over the spectrum in the inset). The metastable state features a oscillatory spacing around 9 ps and a fixed π-phase difference (marked by arrows in bottom panels), resembling the results shown in Fig.5d and Fig.S13.

The simulation results highly agreed with the experiment observations in terms of the key features mentioned above, while the presence of the parallel trapping potentials in experiments unfolded these features in a unique panorama. By finely tuning the cavity parameters, we noticed that the gain filtering effect is of particular importance for the formation of soliton molecules. Under a π-phase difference, the partially overlapped solitons would exert repulsions due to interferometric overlapping, while the gain filtering effect would counter balance the repulsion and result in a stable spacing. In addition, the Kelly sidebands may also play an important role during the soliton molecule formation, which seems to discretize the accessible spacing between the solitons in a bound state. Therefore, unlike the long-range binding of optical solitons in the supramolecular structure[7], the soliton spacing in conventional soliton molecule cannot be freely tuned given a fixed cavity configurations.

We notice that the simulated soliton molecule has an inner-spacing different from that observed in experiments (by $\sim$ 1 ps). This is probably due to the imprecise estimation of cavity parameters, especially the dispersion of higher orders, which may shift the exact location of the Kelly sidebands and therefore leads to a different binding spacing. Some other effects (e.g. asymmetric gain spectrum, higher-order nonlinearity, etc.) should also be added to this model in order further to increase the precision.

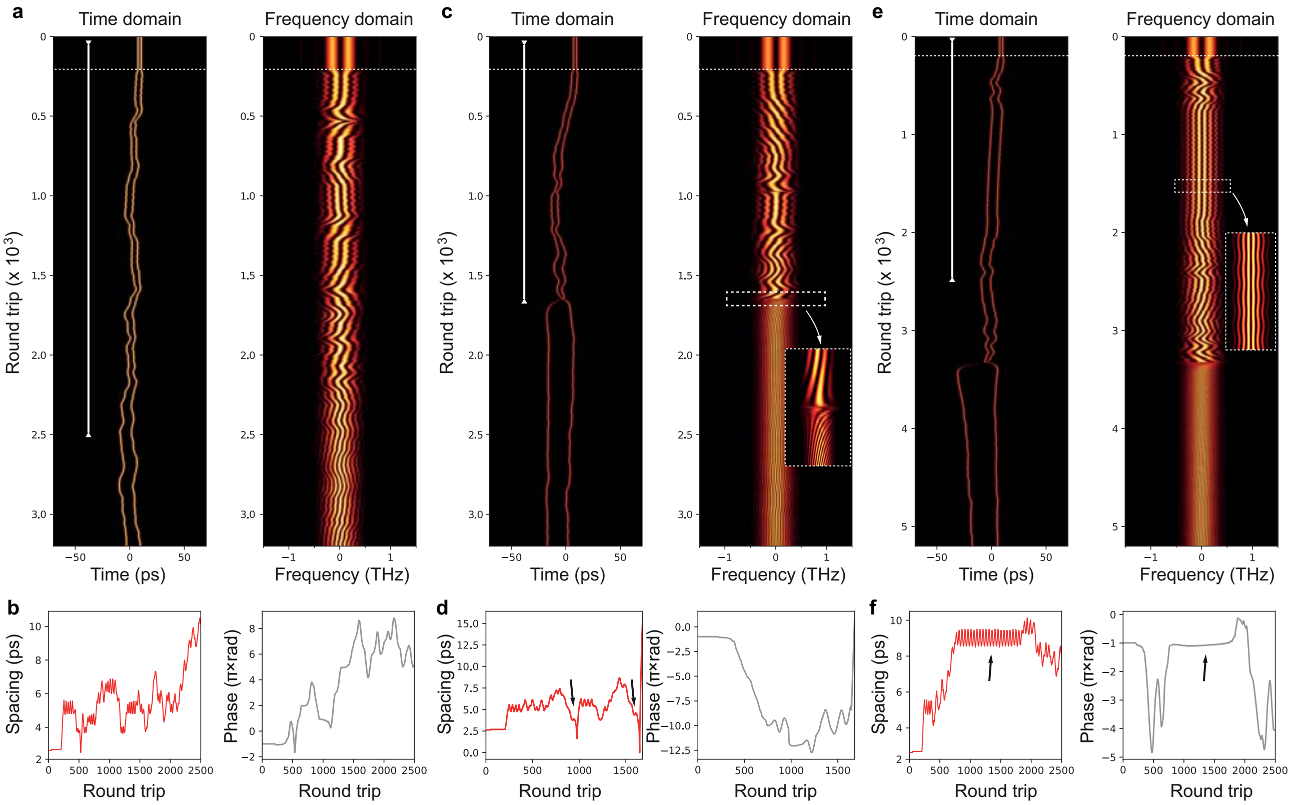

FIG. S17. Three examples of numerical simulation of soliton molecule dissociation initiated by gain perturbation. The initial soliton molecule are stable results of identical cavity parameters, while the perturbations differs by only an order of 10^{-4}. Each example consists of the evolution of soliton motion in time and spectral domain (**a**, **c**, and **e**) together with the corresponding evolution of soliton spacing and relative phase (**b**, **d**, and **f**). The starting of the dissociation induced by gain perturbation is marked by the the horizontal dashed line. The white vertical lines marked the region where the evolutions of soliton spacing and relative phase are plotted.

VII. PARALLEL REACTIONS OF SOLITON-TRIPLET MOLECULES

The synthesis of a soliton-triplet molecule started with long-range bound-states of a soliton-pair molecule and a single-soliton in each reactor. The reaction was imitated by increasing the cavity-loss by 1-dB over a 50-µs rising-edge. The long-range binding then collapsed and the single-soliton went through multiple collisions with the soliton-pair in each reactor, before an effective collision occurred that led to formation of a soliton-triplet (Fig.S18a). The synthesis in 8 consecutive time-slots (out of 160) recorded over the initial 2.5 ms are shown in Fig.S18 b and c with both time-domain sequence and the DFT signal. We can first notice that the trajectories are highly diverse from reactor to reactor with both successful and failed synthesis. We can also notice the discrepancies in group velocities between different solitonic elements during the dynamic process (compared with

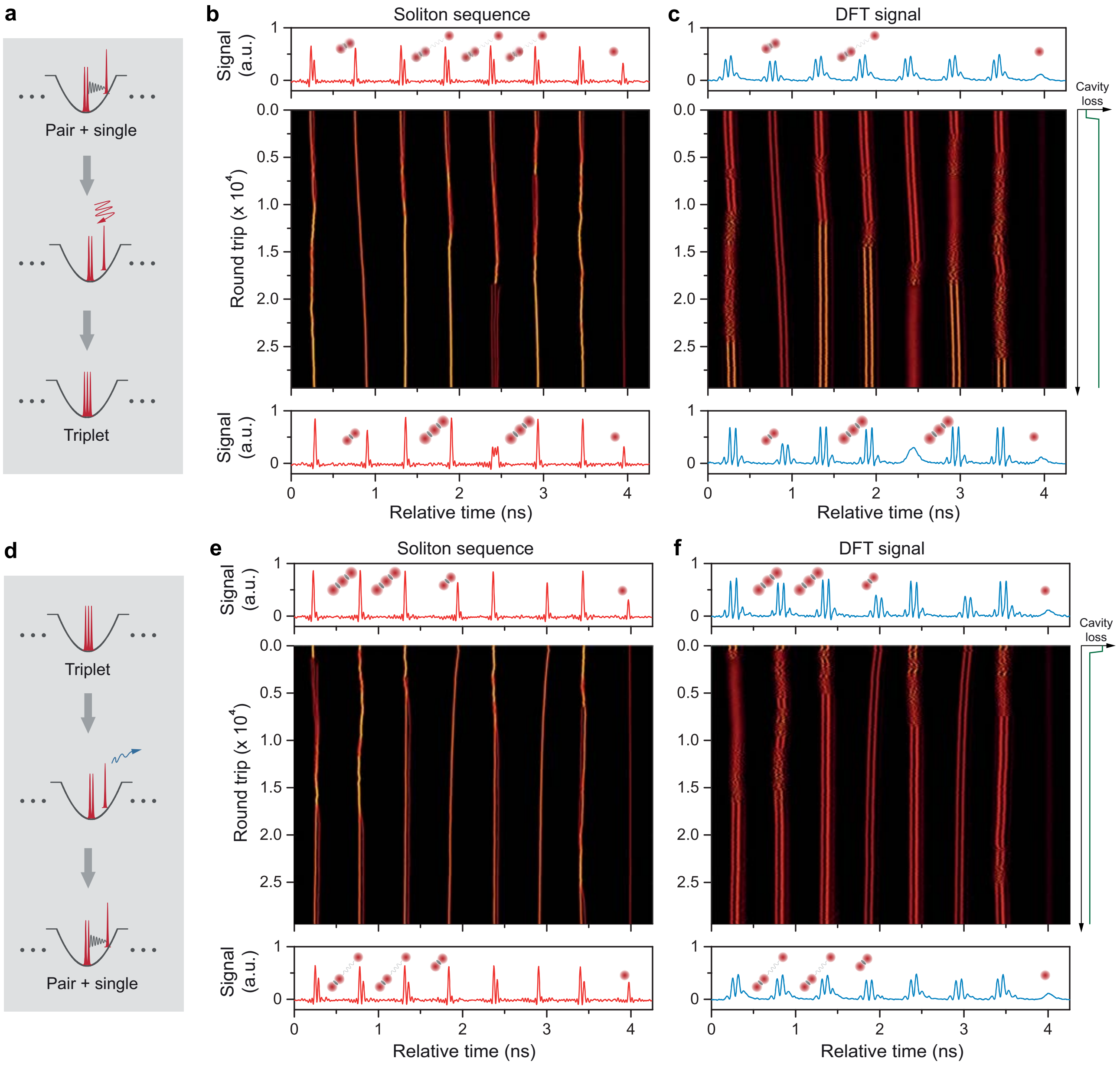

FIG. S18. **a** Schematic of synthesis dynamics. **b** The time-domain soliton sequence in 8 (out of 160) consecutive time-slots recorded over the initial $\sim$ 29000 round-trips (2.5 ms). An abrupt 1-dB increase of cavity loss over a 50-µs edge is applied at the beginning to initiate the reactions. **c** The corresponding DFT signal of the time-domain sequence in **b**. **d** Schematic of a typical dissociation of an soliton-triplet molecule, with one of the molecular bond severed. **e** The time-domain recording of 8 consecutive time-slots (out of 160) over $\sim$ 29000 round trips of soliton-triplet dissociation. A few time-slots contain either a soliton-pair or a single-soliton and exhibit the different group-velocity during the dissociation. **f** The corresponding DFT signal of the time-domain recording in **e**.

the reference time-slots that contain only a soliton-pair or single-soliton). The trapping potentials provided by the optomechanical lattice passively synchronize all these different elements and settle the velocity discrepancies into different trapping positions within each time-slot, avoiding "cross-talks" between the reactors.

The parallel dissociation of soliton-triplet molecules within the parallel reactors are also highly stochastic as we observed in our experiment, although typically most soliton triplets dissociated into a soliton-pair and a single-soliton in the final state (Fig.S18d). We plot the time-domain sequence and the corresponding DFT signal over 8 (out of 160) consecutive time-slots during the initial 2.5 ms of the parallel dissociation (Fig.S18e and f). The reaction was initiated by a 1-dB decrease of the cavity loss over a 50-µs falling-edge. Similar to the case in synthesis, the group-velocity discrepancies between different solitonic elements have also been noticed. Moreover, apart from the smooth dissociation shown in Fig.6, some radical break-down of the soliton-triplet molecules that led to extinctions of the constituting solitons have also be observed, as shown in Fig.S19. These phenomena are similar to the soliton repulsions in two-soliton reactions shown in Fig.5 and Fig.S11.

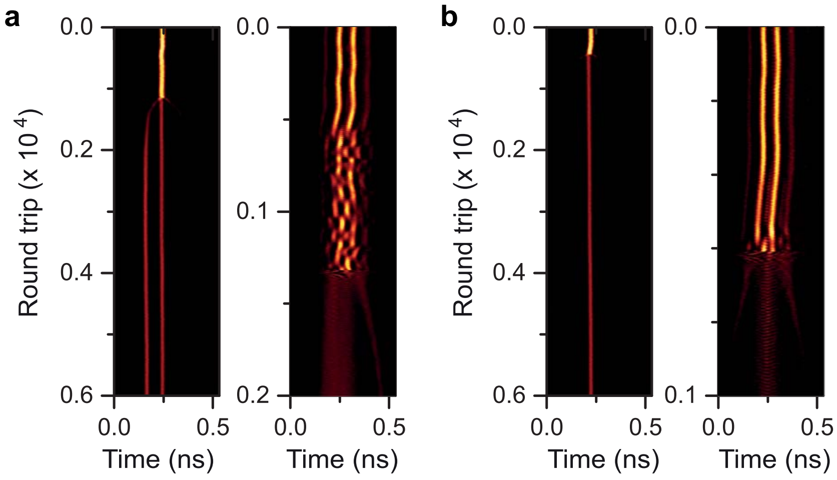

FIG. S19. Dissociation of a soliton triplet that end up with losing one (**a**) and two (**b**) of the reacting solitons after a radical repulsion. Left-panels are time-domain evolutions while the right-panels are the DFT signal. The two examples are chosen from the parallel reaction same as in Fig.6

VIII. MECHANISM OF INDIVIDUAL CONTROL

For synthesis of soliton molecule in selected time-slots, the addressing pulse pattern is set to be synchronized with intra-cavity soliton in terms of the cavity round-trip frequency. Each addressing pulse can then constantly overlap with the target time-slot without relative drifting. The two solitons, due to their large spacing, would experience different nonlinear

index induced by XPM when riding at different position of the addressing pulse, which leads to relative motion of two solitons that appeared as attraction between the two solitons. Meanwhile, the repulsive force between the solitons due to dispersive wave perturbation could also be perturbed by the overlapping of the addressing pulses. Consequently, the effective attraction between the two solitons exceeds their repulsion and the two solitons start to move towards each other, until the spacing is small enough that their direct, phase-sensitive interactions start to take place (see Fig.S20a).

In the example shown Fig.7b, such collision ended up with successful formation of soliton-pair molecule. while in most other time-slots, however, the collisions failed to establish a molecular bond, and the two solitons repel each other again into the long-range bound states. One example is shown in Fig.S20b. This is an fully expected result, considering that multiple collisions were usually required for soliton molecule synthesis under global control (e.g. see Fig.2), while the initial phase relation between the interacting solitons varied from time-slot to time-slot.

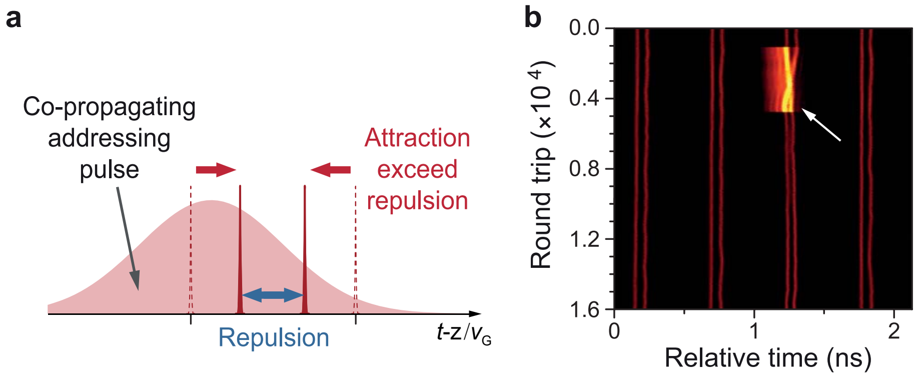

FIG. S20. **a** The overlapping of the addressing pulses with the two solitons causes additional attraction that exceeds the repulsions between them, leading to soliton collisions. **b** Time-domain recording of a few time-slots during the individual control experiment (same as the one shown in Fig.7b). One time-slot (marked by white arrow) that host a long-range double-soliton was perturbed by addressing pulses that constantly overlap with it, leading to however an ineffective collision.

For dissociation of soliton molecules in selected time-slots, we tried in experiments different sets of parameters for the addressing pulses in order to achieve fast dissociation. We conclude empirically that the most effective method seems to be launching the addressing pulses with a slightly asynchronized repetition rate (in our case $\sim$ 20 Hz). The addressing pulses would then "walk through" the target soliton-pair molecules (Fig.S21a). The dissociation is probably due to a mutual frequency shift caused by the traversing pulses, which turned the phase-relation of two solitons from π towards to 2π (or 0). Then the force between the two solitons became net attraction[9], leading to the sharp drop of soliton spacing. As a consequence, a strong repulsion is triggered, similar to the cases shown in Fig.5e (or

Fig.S16), leading to fast dissociation of the molecule, and the establishment of long-range binding. This process can be revealed from the DFT signal over the perturbed region shown in Fig.S21b and the retrieved trajectories in Fig.S21b. The soliton spacing is first squeezed to ~ 2 ps while the relative phase shift from π towards to 2π, leading to a radical repelling and then immediate dissociation.

The induced mutual frequency-shift of the solitons could be caused by multiple effects. Firstly, the traversing pulses can cause a soliton-dragging effect via a varying XPM (see Refs[13,14]), leading to a soliton frequency shift that obeys $\Delta\omega \propto |\partial I_a/\partial t|$, where I_a is the local intensity of addressing pulse profile at the soliton molecule. Secondly, the time-variant XPM might also influence the NPR of the overlapped solitons, leading to a time-variant loss of them at the in-cavity polariser, which is then translated into a frequency shift through the gain filtering effect. The significance of this traversing-pulse method is that it has turned a probabilistic event —the radical soliton repulsion observed only in the stochastic trajectories of soliton reaction (see Fig.5e) —into a deterministic event that can be triggered on demand in our experiments.

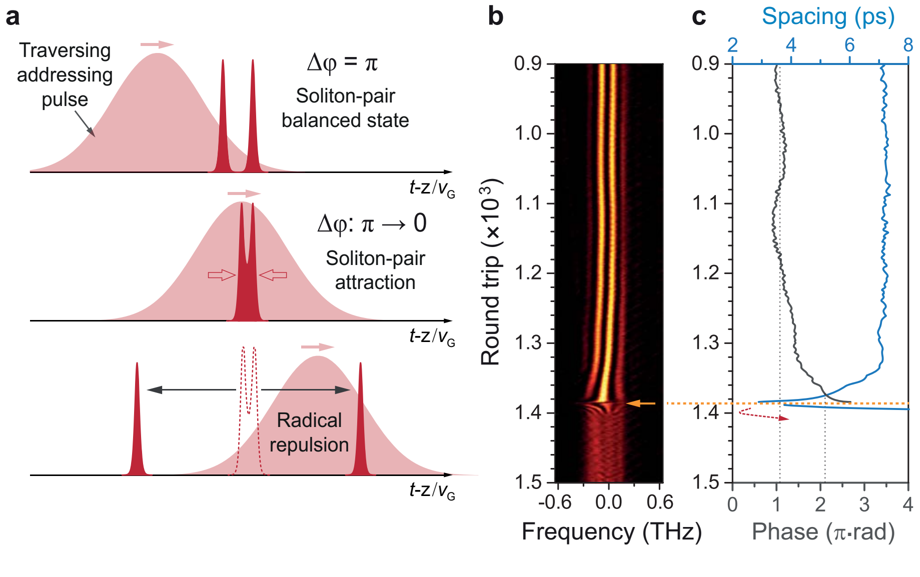

FIG. S21. **a** Schematic of the dissociation mechanism through addressing-pulse perturbation. The addressing pulses traverse across the target soliton-pair, causing a flipping of the phase-relation (from out-of-phase to in-phase) and consequently narrowing of the soliton spacing. Then a radical repulsion occurred which quickly push the two solitons away from each other. **b** DFT signal and **c** the retrieved spacing and phase-relation between the two solitons during the dissociation. A radical repulsion occurred (marked by the yellow arrow) before the two solitons completely dissociate.

REFERENCES

[1]M. Pang, X. Jiang, W. He, G. K. L. Wong, G. Onishchukov, N. Y. Joly, G. Ahmed, C. R. Menyuk, and P. St.J. Russell, "Stable subpicosecond soliton fiber laser passively mode-locked by gigahertz acoustic resonance in photonic crystal fiber core," Optica **2**, 339 (2015).

[2]W. He, M. Pang, and P. St.J. Russell, "Wideband-tunable soliton fiber laser mode-locked at 1.88 GHz by optoacoustic interactions in solid-core pcf," Optics Express **23**, 24945–24954 (2015).

[3]M. Pang, W. He, X. Jiang, and P. St.J. Russell, "All-optical bit storage in a fibre laser by optomechanically bound states of solitons," Nature Photonics **10**, 454–458 (2016).

[4]W. He, M. Pang, C. R. Menyuk, and P. St.J. Russell, "Sub-100-fs 1.87 GHz mode-locked fiber laser using stretched-soliton effects," Optica **3**, 1366 (2016).

[5]M. Hofer, M. E. Fermann, F. Haberl, M. H. Ober, and A. J. Schmidt, "Mode locking with cross-phase and self-phase modulation," Optics Letters **16**, 502–504 (1991).

[6]C.-J. J. Chen, P. K. A. Wai, and C. R. Menyuk, "Soliton fiber ring laser," Optics Letters **17**, 417 (1992).

[7]W. He, M. Pang, D. H. Yeh, J. Huang, C. R. Menyuk, and P. St.J. Russell, "Formation of optical supramolecular structures in a fibre laser by tailoring long-range soliton interactions," Nature Communications **10**, 5756 (2019).

[8]J. Igbonacho, K. Nithyanandan, K. Krupa, P. T. Dinda, P. Grelu, and A. B. Moubissi, "Dynamics of distorted and undistorted soliton molecules in a mode-locked fiber laser," Physical Review A **99**, 063824 (2019).

[9]F. M. Mitschke and L. F. Mollenauer, "Experimental observation of interaction forces between solitons in optical fibers," Optics Letters **12**, 355–357 (1987).

[10]F. M. Mitschke and L. F. Mollenauer, "Discovery of the soliton self-frequency shift," Optics Letters **11**, 659 (1986).

[11]K. Tamura, E. P. Ippen, H. A. Haus, and L. E. Nelson, "77-fs pulse generation from a stretched-pulse mode-locked all-fiberring laser," Optics Letters **18**, 1080–1082 (1993).

[12]W. Weng, R. Bouchand, E. Lucas, and T. J. Kippenberg, "Polychromatic cherenkov radiation induced group velocity symmetry breaking in counterpropagating dissipative kerr solitons," Physical Review Letters **123**, 253902 (2019).

[13]M. N. Islam, J. R. Simpson, H. T. Shang, L. F. Mollenauer, and R. H. Stolen, "Cross-phase modulation in optical fibers," Optics Letters **12**, 625 (1987).

[14]M. N. Islam, C. R. Menyuk, C.-J. Chen, and C. E. Soccolich, "Chirp mechanisms in soliton-dragging logic gates," Optics Letters **16**, 214 (1991).
